# Metformin Use in Relation to Clinical Outcomes and Hyperinflammatory Syndrome Among COVID-19 Patients With Type 2 Diabetes: A Propensity Score Analysis of a Territory-Wide Cohort

**DOI:** 10.3389/fendo.2022.810914

**Published:** 2022-03-07

**Authors:** Carlos K. H. Wong, David T. W. Lui, Angel Y. C. Lui, Marshall C. H. Low, Ashley C. Y. Kwok, Kristy T. K. Lau, Ivan C. H. Au, Xi Xiong, Matthew S. H. Chung, Eric H. Y. Lau, Benjamin J. Cowling

**Affiliations:** ^1^ Centre for Safe Medication Practice and Research, Department of Pharmacology and Pharmacy, Li Ka Shing (LKS) Faculty of Medicine, The University of Hong Kong, Hong Kong SAR, China; ^2^ Department of Family Medicine and Primary Care, Li Ka Shing (LKS) Faculty of Medicine, The University of Hong Kong, Hong Kong SAR, China; ^3^ Laboratory of Data Discovery for Health Limited, Hong Kong SAR, China; ^4^ Division of Endocrinology and Metabolism, Department of Medicine, Li Ka Shing (LKS) Faculty of Medicine, The University of Hong Kong, Hong Kong SAR, China; ^5^ World Health Organization (WHO) Collaborating Centre for Infectious Disease Epidemiology and Control, School of Public Health, Li Ka Shing (LKS) Faculty of Medicine, The University of Hong Kong, Hong Kong SAR, China

**Keywords:** type 2 diabetes, metformin, COVID-19, SARS-CoV-2, in-hospital mortality, hyperinflammatory syndrome

## Abstract

**Aim:**

This study was conducted in order to evaluate the association between metformin use and clinical outcomes in type 2 diabetes mellitus (T2DM) patients hospitalized with coronavirus disease 2019 (COVID-19).

**Methods:**

Patients with T2DM with confirmed diagnosis of COVID-19 and admitted between January 21, 2020, and January 31, 2021 in Hong Kong were identified in our cohort. Exposure was defined as metformin use within 90 days prior to admission until hospital discharge for COVID-19. Primary outcome was defined as clinical improvement of ≥1 point on the WHO Clinical Progression Scale (CPS). Other outcomes were hospital discharge, recovery, in-hospital death, acidosis, hyperinflammatory syndrome, length of hospitalization, and changes in WHO CPS score.

**Results:**

Metformin use was associated with greater odds of clinical improvement (OR = 2.74, *p* = 0.009), hospital discharge (OR = 2.26, *p* = 0.009), and recovery (OR = 2.54, *p* = 0.005), in addition to lower odds of hyperinflammatory syndrome (OR = 0.71, *p* = 0.021) and death (OR = 0.41, *p* = 0.010) than control. Patients on metformin treatment had a shorter hospital stay (−2.76 days, *p* = 0.017) than their control counterparts. The average WHO CPS scores were significantly lower in metformin users than non-users since day 15 (*p* < 0.001). However, metformin use was associated with higher odds of acidosis.

**Conclusions:**

Metformin use was associated with lower mortality and lower odds for hyperinflammatory syndrome. This provides additional insights into the potential mechanisms of the benefits of metformin use in T2DM patients with COVID-19.

## Introduction

The coronavirus disease 2019 (COVID-19) pandemic, caused by severe acute respiratory syndrome coronavirus 2 (SARS-CoV-2), has infected over 208 million people globally as of August 19, 2021 (1). Type 2 diabetes mellitus (T2DM) is associated with worse clinical outcomes in COVID-19 patients, such as requirement of intensive care unit (ICU) admission and all-cause mortality (2–4). Patients with diabetes are more vulnerable, owing to the associated hyperglycemia causing dysregulated innate immunity and the associated low-grade chronic inflammation which increases the likelihood of cytokine storms in COVID-19, hence leading to adverse clinical outcomes (3, 5, 6).

As metformin is the first-line antidiabetic medication, it is important to understand the benefits and risks of its use among COVID-19 patients. While retrospective cohort studies have mostly revealed better outcomes associated with metformin use among COVID-19 patients with diabetes, such as intubation, ICU admission, and mortality (7–9), clinical data providing mechanistic links are limited. The potential beneficial effects of metformin probably extend beyond its glucose-lowering effect. In fact, metformin has also been proposed to possess antiviral effects and influence the level of inflammatory cytokine production (6). Nevertheless, one has to consider the potential adverse effects due to metformin use. Lactic acidosis is among one of the most commonly raised concerns (4, 10–12), which may be relevant in patients with renal impairment despite being extremely rare in clinical settings (10).

Recommendations differ regarding metformin use in COVID-19 patients: while the Joint British Diabetes Societies for inpatient care has proposed to continue metformin use, possibly due to being associated with better clinical outcomes (13), one published review article suggested stopping metformin in case of acute illness and respiratory distress given the concerns about risk of acidosis (11). Hence, a more in-depth evaluation of the potential effects of metformin use in a cohort of COVID-19 patients with diabetes will shed light on this issue.

Hence, we initiated this analysis of a territory-wide cohort of COVID-19 patients with T2DM to evaluate the impact of metformin use in relation to clinical outcomes, hyperinflammatory syndrome, and viral loads.

## Materials and Methods

### Data Source and Study Population

Our data were extracted from a territory-wide cohort of patients with anonymized electronic health records provided by the Hong Kong Hospital Authority (HA). The public health ordinance in Hong Kong required all patients diagnosed with COVID-19 to be isolated in public hospitals, including those detected on contact tracing and the universal community testing program, regardless of symptoms. All COVID-19 cases would be captured as the HA is the only public-funded healthcare provider managing COVID-19 patients in Hong Kong.

In this study, all patients who had been diagnosed with T2DM and were tested positive for COVID-19 were included, if they were admitted to public hospitals between January 21, 2020, and January 31, 2021 in Hong Kong SAR, China. COVID-19 was confirmed by positive SARS-CoV-2 viral nucleic acid detected using real-time reverse transcription-polymerase chain reaction (RT-PCR) assay, performed by the Public Health Laboratory under the Department of Health. Patients with T2DM were captured using the International Classification of Primary Care, Version 2 (ICPC2) code T90 or the International Statistical Classification of Diseases and Related Health Problems, 9th Revision, Clinical Modification (ICD-9-CM) codes 250.x0 or 250.x2. This cohort has been used for studying pharmacoepidemiology of drug treatment for COVID-19 (14).

Each eligible patient was observed from the date of hospital admission (baseline, day 0) to the date of in-hospital death, hospital discharge, or data cutoff date (April 30, 2021), whichever came first.

### Definition of Metformin Exposure

Patients were classified as metformin users and non-users according to their exposure to metformin. Metformin users were patients who had received metformin from 90 days prior to admission to the day of discharge due to SARS-CoV-2 infection. T2DM patients who had not received or used metformin during the stated period were categorized as non-users.

### Definition of Covariates

Pre-existing comorbidity profile was represented by the Charlson comorbidity index; hypertension; chronic lung, heart, and kidney diseases; liver disease; malignancy; and obesity and was captured based on ICD-9-CM diagnosis codes. Long-term medications taken by the patients in the past 3 years included angiotensin-converting enzyme inhibitors (ACEI) or angiotensin receptor blockers (ARB), anticoagulants, antiplatelets, lipid-lowering agents, and non-steroidal anti-inflammatory drugs (NSAID). The use of concomitant antidiabetic agents from 90 days prior to admission to hospital discharge was also recorded [namely, glucagon-like peptide 1 receptor agonists (GLP1RA), insulin, sulfonylureas (SU), thiazolidinediones (TZD), acarbose, dipeptidyl peptidase 4 inhibitors (DPP4i), and sodium-glucose cotransporter-2 inhibitors (SGLT2i)].

A score of baseline COVID-19 severity was assigned based on the WHO Clinical Progression Scale (15). Disease severity was categorized into 1) not requiring any oxygen therapy, score 4; 2) requiring supplemental oxygen without ventilation, score 5–6; and 3) requiring mechanical ventilation, score 7–9 (15). COVID-19 drug treatments were documented (remdesivir, interferon-β-1b, dexamethasone, and tocilizumab). The clinical condition of each patient at baseline was recorded, including the need for ICU admission, admission *via* the emergency department, initiation of extracorporeal membrane oxygenation (ECMO), and dialysis, as well as the occurrence of acute respiratory distress syndrome (ARDS) and hyperinflammatory syndrome [as defined by Webb et al., which consists of macrophage activation, hematological dysfunction, coagulopathy, and hepatic inflammation (16)]. A comprehensive panel of hematological and biochemical laboratory parameters was obtained, coupled with regular assessments during COVID-19. These included white blood cell, neutrophil, lymphocyte, platelet, lactate dehydrogenase, creatine kinase, total bilirubin, C-reactive protein, estimated glomerular filtration rate (eGFR), alkaline phosphatase (ALP), alanine transaminase (ALT), hemoglobin, prothrombin time, random glucose, and glycated hemoglobin (HbA1c). The presence of SARS-CoV-2 was confirmed in all patients by reverse transcription-polymerase chain reaction (RT-PCR) from the nasopharyngeal swab or deep throat saliva. A standardized real-time RT-PCR assay was used to detect the E gene of SARS-CoV-2. The cycle threshold (Ct) value represents the number of cycles required for a gene target or a PCR product to be detected. While viral loads were not directly measured with a dedicated quantitative RT-PCR assay in this analysis, studies have shown a good correlation between Ct values and SARS-CoV-2 viral loads, such that the lower the Ct values, the higher the viral loads (17).

### Definition of Outcomes

The primary outcome of this study was clinical improvement, defined as a reduction of at least one point on the WHO Clinical Progression Scale.

The secondary outcomes were as follows: i) hospital discharge; ii) recovery without the need for oxygen therapy; iii) in-hospital death; iv) incidence of acidosis; v) incidence of hyperinflammatory syndrome [defined by Webb et al., including macrophage activation, hematological dysfunction, coagulopathy, and hepatic inflammation (16)]; vi) length of hospitalization; vii) clinical status as measured by the WHO Clinical Progression Scale scores on days 0, 7, 15, 30, 60, and 90; viii) WHO Clinical Progression Scale scores on days 0, 7, 15, 30, 60, and 90; ix) Ct values on days 0, 7, and 15; x) proportion of patients with IgG antibody on days 3, 7, and 15; and xi) cumulative direct medical costs incurred on days 0, 7, 15, 30, 60, and 90. The cumulative healthcare cost was calculated based on the unit cost of medication and healthcare services sourced from the Hong Kong SAR Government Gazette and the Hospital Authority (**Supplementary Table 1**).

### Data Analysis

Descriptive statistics of baseline characteristics between the treatment and control groups were presented as mean and standard deviation for continuous variables and count and proportion for categorical variables.

Data completion rates of patient characteristics at baseline are shown in **Supplementary Table 2**. For the missing baseline covariates upon admission, multiple imputation by chained equations (MICE) was employed in the treatment and control groups. Each missing value of laboratory data was imputed 20 times using other variables that might have an impact on the study outcomes.

The propensity scores of each patient in the cohort with the aforementioned covariates were calculated with a logistic regression model. To minimize the outcome bias caused by variations in baseline characteristics, inverse probability of treatment weighting (IPTW) was then implemented to balance the covariates between groups using the calculated propensity scores. After propensity-score weighting, the balance of baseline covariates between treatment groups would be further evaluated using the standardized mean difference (SMD). SMDs ≤0.2 indicated a sufficient balance between groups.

A multivariable logistic regression model weighted by IPTW was adopted in order to estimate the effects of exposure on binary outcomes in odds ratios (OR) and their 95% confidence intervals (CI). The effects of exposure on Ct values, hospital length of stay among survivors, and healthcare costs were all estimated using the multivariable linear regression model weighted by IPTW.

Lastly, sensitivity and subgroup analyses were conducted. Sensitivity analyses included removing hospital discharge as a censoring criterion and limiting the follow-up period to a maximum of 90 days. Subgroup analyses were done on several patient subgroups, namely, age (≤65 and >65 years), sex, initiation of invasive mechanical ventilation or ECMO, ICU admission, any concomitant use of other medications (insulin, SU, remdesivir, interferon-β-1b, and dexamethasone), in-hospital use of metformin without prior metformin use, and without concomitant use of insulin.

All statistical analyses were performed using STATA Version 16 (StataCorp LP, College Station, TX, USA). *p*-value <0.05 was considered statistically significant.

## Results

In total, 1,214 T2DM patients were admitted for confirmed COVID-19 between January 21, 2020, and January31, 2021 in Hong Kong, while 786 patients (64.7%) were metformin users. After multiple imputation and propensity score weighting, all patient characteristics were balanced between groups at baseline with SMDs ≤0.2 (**Table 1**). **Supplementary Figure 1** demonstrates that propensity score density was highly overlapped after propensity score weighting. Overall, the median follow-up period of this patient cohort was 16 days with 30,035 person-days. The incidence rates of outcome events by exposure and control groups are presented in **Supplementary Table 3**. There were 97.1% of patients achieving clinical improvement in the metformin group and 80.4% in the control group.

**Table 1:** Baseline characteristics of T2DM patients hospitalized with COVID-19 by exposure to metformin after multiple imputation and propensity score weighting.

	Before weighting						After weighting					
Baseline characteristics	Metformin (*n* = 786)		Control (*n* = 428)		SMD	Metformin (*n* = 786)			Control (*n* = 428)		SMD
	*N* / mean	% / SD	*N* / mean	% / SD		*N* / mean		% / SD	*N* / mean	% / SD	
Age, years^a^	64.0	12.2	67.5	13.7	0.27	65.8		12.5	67.2	12.7	0.12
≤65	418	(53.2%)	183	(42.8%)	0.21	(47.6%)			(45.6%)		0.04
>65	368	(46.8%)	245	(57.2%)		(52.4%)			(54.4%)		
Sex					0.03						0.01
Male	423	(53.8%)	237	(55.4%)		(53.7%)			(53.3%)		
Female	363	(46.2%)	191	(44.6%)		(46.3%)			(46.7%)		
Pre-existing comorbidities											
Charlson index^a^ ^,^ ^b^	5.0	1.6	5.9	2.2	0.52	5.4		1.8	5.6	1.9	0.07
1–4	264	(33.6%)	92	(21.6%)		(26.9%)			(23.2%)		
5-6	415	(52.8%)	198	(46.4%)		(50.8%)			(52.3%)		
7-14	107	(13.6%)	137	(32.1%)		(22.3%)			(24.5%)		
Hypertension	587	(74.7%)	341	(79.7%)	0.12	(78.0%)			(77.7%)		0.01
Chronic lung disease	55	(7.0%)	72	(16.8%)	0.31	(12.3%)			(10.4%)		0.06
Chronic heart disease	107	(13.6%)	78	(18.2%)	0.13	(14.6%)			(15.4%)		0.02
Chronic kidney disease	63	(8.0%)	95	(22.2%)	0.40	(13.4%)			(15.0%)		0.04
Liver disease	90	(11.5%)	63	(14.7%)	0.10	(16.8%)			(14.4%)		0.06
Malignancy	20	(2.5%)	21	(4.9%)	0.12	(3.8%)			(4.6%)		0.04
Obesity	93	(11.8%)	48	(11.2%)	0.02	(12.2%)			(12.3%)		0.01
Preadmission or in-hospital use											
Metformin	786	(100.0%)	0	(0.0%)	NA	(100.0%)			(0.0%)		NA
GLP1RA	8	(1.0%)	2	(0.5%)	0.06	(0.7%)			(0.3%)		0.07
Insulin	417	(53.1%)	277	(64.7%)	0.24	(54.4%)			(60.1%)		0.11
Oral antidiabetic drugs											
SU	373	(47.5%)	57	(13.3%)	0.80	(36.5%)			(38.4%)		0.04
TZD	70	(8.9%)	12	(2.8%)	0.26	(6.5%)			(5.3%)		0.05
Acarbose	2	(0.3%)	2	(0.5%)	0.04	(0.2%)			(0.5%)		0.04
DPP4i	74	(9.4%)	33	(7.7%)	0.06	(7.3%)			(7.6%)		0.01
SGLT2i	26	(3.3%)	3	(0.7%)	0.19	(2.3%)			(2.0%)		0.02
Long-term medications											
ACEI/ARB	395	(50.3%)	152	(35.5%)	0.30	(45.3%)			(48.9%)		0.07
Anticoagulant	219	(27.9%)	215	(50.2%)	0.47	(36.0%)			(35.7%)		0.01
Antiplatelet	167	(21.2%)	106	(24.8%)	0.08	(20.9%)			(26.4%)		0.13
Lipid-lowering agent	488	(62.1%)	185	(43.2%)	0.38	(53.1%)			(60.3%)		0.15
NSAID	185	(23.5%)	114	(26.6%)	0.07	(22.7%)			(28.6%)		0.14
In-hospital COVID-19 drug use											
Remdesivir	207	(26.3%)	121	(28.3%)	0.04	(27.8%)			(24.7%)		0.07
Interferon-β-1b	503	(64.0%)	306	(71.5%)	0.16	(64.8%)			(68.2%)		0.07
Dexamethasone	341	(43.4%)	255	(59.6%)	0.33	(49.8%)			(47.7%)		0.04
Tocilizumab	41	(5.2%)	66	(15.4%)	0.34	(9.9%)			(10.3%)		0.01
ECMO	4	(0.5%)	8	(1.9%)	0.13	(0.5%)			(0.7%)		0.03
Dialysis	17	(2.2%)	45	(10.5%)	0.35	(3.2%)			(6.4%)		0.15
ICU admission on admission	104	(13.2%)	150	(35.0%)	0.53	(20.2%)			(19.2%)		0.03
Admission *via* the emergency department	368	(46.8%)	236	(55.1%)	0.17	(51.2%)			(50.8%)		0.01
Clinical severity on admission by WHO Clinical Progression Scale											
Score (range 0–10)^a^	4.4	0.8	4.8	1.1	0.53	4.6		1.0	4.5	0.9	0.07
No oxygen therapy (score 4)	647	(82.3%)	255	(59.6%)		(75.0%)			(76.2%)		
Supplemental oxygen without ventilation (score 5–6)	133	(16.9%)	164	(38.3%)		(21.9%)			(23.0%)		
Mechanical ventilation (score 7–9)	6	(0.8%)	9	(2.1%)		(3.1%)			(0.8%)		
ARDS	13	(1.7%)	16	(3.7%)	0.13	(2.3%)			(3.4%)		0.06
Macrophage activation	0	(0.0%)	0	(0.0%)	NA	(0.0%)			(0.0%)		NA
Haematological dysfunction	41	(5.2%)	40	(9.3%)	0.16	(8.8%)			(6.1%)		0.10
Coagulopathy	3	(0.4%)	3	(0.7%)	0.04	(0.7%)			(0.3%)		0.05
Hepatic inflammation	48	(6.1%)	45	(10.5%)	0.16	(7.8%)			(6.3%)		0.06
Hyperinflammatory syndrome	81	(10.3%)	75	(17.5%)	0.21	(14.6%)			(10.2%)		0.13
Laboratory parameters (normal range)^a^											
White blood cell, ×10^9^/L (3.7–9.2 × 10^9^/L)	5.9	2.1	6.4	3.0	0.20	6.1		2.4	6.3	2.8	0.05
Neutrophil, ×10^9^/L (1.7–5.8 × 10^9^/L)	4.0	1.9	4.6	2.8	0.29	4.3		2.4	4.2	2.3	0.05
Lymphocyte, ×10^9^/L (1.0–3.1 × 10^9^/L)	1.2	0.7	1.1	0.8	0.18	1.2		0.7	1.4	1.7	0.16
Platelet, ×10^9^/L (145–370 × 10^9^/L)	208.6	75.8	199.4	77.9	0.12	200.5		76.5	207.5	82.2	0.09
Lactate dehydrogenase, U/L (110–210 U/L)	251.1	109.7	301.7	164.2	0.39	272.1		133.2	268.5	129.0	0.03
Creatine kinase, U/L (26–192 U/L)	186.8	304.1	249.9	503.3	0.16	208.0		342.2	247.9	384.6	0.11
Total bilirubin, μmol/L (5–27 μmol/L)	8.8	6.6	9.9	7.3	0.15	10.2		14.2	9.5	6.0	0.06
C-reactive protein, mg/L (<5 mg/L)	37.2	51.6	50.4	64.3	0.23	41.4		53.8	40.3	57.9	0.02
Cycle threshold value, cycle	22.8	6.4	22.0	6.3	0.12	22.5		6.3	22.2	6.1	0.04
eGFR, ml/min/1.73 m^2^ (>90 ml/min/1.73 m^2^)	106.4	88.2	99.2	103.4	0.08	102.2		85.5	96.7	73.6	0.07
ALP, U/L (30–120 U/L)	72.6	25.7	80.5	41.3	0.25	72.9		26.8	75.3	30.2	0.08
ALT, U/L (<46.5 U/L)	37.2	41.7	36.8	28.3	0.01	39.4		45.4	37.6	28.5	0.04
Hemoglobin, g/dl (13.4–17.1 g/dl)	13.2	1.6	12.8	1.9	0.24	13.0		1.7	13.1	1.9	0.04
Prothrombin time, s (10–14 s)	11.8	3.0	12.9	4.8	0.28	12.2		3.0	12.1	3.8	0.04
Random glucose, mmol/L (3–11 mmol/L)	10.2	4.9	8.3	4.6	0.38	9.1		5.4	9.8	5.7	0.12
HbA1c, % (4.8%–6.0%)	8.0	2.4	6.5	2.3	0.63	7.3		2.7	7.7	2.5	0.14

SMD of <0.2 indicates covariate balance between metformin and control groups.

ACEI, angiotensin-converting enzyme inhibitor; ALP, alkaline phosphatase; ALT, alanine transaminase; ARB, angiotensin receptor blocker; ARDS, acute respiratory distress syndrome; COVID-19, coronavirus disease 2019; DPP4i, dipeptidyl peptidase 4 inhibitor; ECMO, extracorporeal membrane oxygenation; eGFR, estimated glomerular filtration rate; GLP1RA, glucagon-like peptide 1 receptor agonist; HbA1c, glycated haemoglobin; ICU, intensive care unit; NA, not applicable; NSAID, non-steroidal anti-inflammatory drug; SD, standard deviation; SGLT2i, sodium-glucose cotransporter-2 inhibitor; SMD, standardized mean difference; SU, sulfonylurea; TZD, thiazolidinedione; T2DM, type 2 diabetes mellitus.

a Age, Charlson index, clinical severity, and laboratory parameters on admission are presented as mean ± SD.

b The calculation of Charlson index does not include acquired immune deficiency syndrome (AIDS).

**Table 2** summarizes the rate of outcomes among metformin users and non-users. Metformin use was associated with significantly higher odds of clinical improvement compared with control (OR: 2.74, 95% CI 1.31 to 5.71, *p* = 0.009), in addition to hospital discharge (OR: 2.26, 95% CI 1.24 to 4.12, *p* = 0.009) and recovery (OR: 2.54, 95% CI 1.34 to 4.80, *p* = 0.005). Besides, metformin users had a significantly shorter length of hospital stay compared with their control counterparts (−2.76 days, 95% CI −5.02 to −0.51, *p* = 0.017). While the odds of acidosis were significantly higher among metformin users (OR: 6.82, 95% CI 2.56 to 18.18, *p* < 0.001), they had significantly lower odds of in-hospital death (OR: 0.41, 95% CI 0.22 to 0.80, *p* = 0.010) and hyperinflammatory syndrome (OR: 0.71, 95% CI 0.53 to 0.95, *p* = 0.021) compared with control. Regarding the criteria for hyperinflammatory syndrome with reference to Webb et al., metformin use was associated with lower odds of macrophage activation (OR: 0.74, 95% CI 0.59 to 0.93, *p* = 0.009) and hepatic inflammation (OR: 0.50, 95% CI 0.38 to 0.65, *p* < 0.001) compared with control. Trends toward a reduction in the odds of hematological dysfunction and coagulopathy were also observed among metformin users. The results of sensitivity were generally comparable to those of the main analysis (**Supplementary Table 4**).

**Table 2:** Comparison of the odds of clinical improvement, in-hospital death, acidosis, and hyperinflammatory syndrome between metformin and control groups.

	Before weighting		After weighting		
	Metformin	Control	Metformin vs. control		
Outcomes	% (*N*)	% (*N*)	OR^a^	95% CI	*p*-value
Clinical improvement on WHO clinical progression scale by ≥1 score	97.1% (786)	80.4% (428)	2.74	(1.31, 5.71)	0.009
Hospital discharge (score ≤ 3)	95.7% (786)	76.6% (428)	2.26	(1.24, 4.12)	0.009
Recovery (score ≤ 4)	85.6% (139)	60.1% (173)	2.54	(1.34, 4.80)	0.005
					
Outcomes	% (*N*)	% (*N*)	OR^b^	95% CI	*p*-value
In-hospital death	3.6% (786)	20.3% (428)	0.41	(0.22, 0.80)	0.010
Acidosis	1.1% (786)	0.9% (428)	6.82	(2.56, 18.18)	<0.001
Hyperinflammatory syndrome	48.8% (705)	64.3% (353)	0.71	(0.53, 0.95)	0.021
Macrophage activation	30.4% (786)	45.1% (428)	0.74	(0.59, 0.93)	0.009
Hematological dysfunction	27.5% (745)	53.4% (388)	0.77	(0.55, 1.10)	0.142
Coagulopathy	6.1% (783)	15.8% (425)	0.81	(0.58, 1.14)	0.234
Hepatic inflammation	23.0% (738)	47.3% (383)	0.50	(0.38, 0.65)	<0.001

CI, confidence interval; OR, odds ratio.

a OR >1 (or <1) indicates that the metformin group was associated with higher (lower) odds of clinical improvement, hospital discharge, or recovery, compared with the control group.

b OR >1 (or <1) indicates that the metformin group was associated with higher (lower) risk of in-hospital death, acidosis, and hyperinflammatory syndrome compared with the control group.

**Figure 1:**
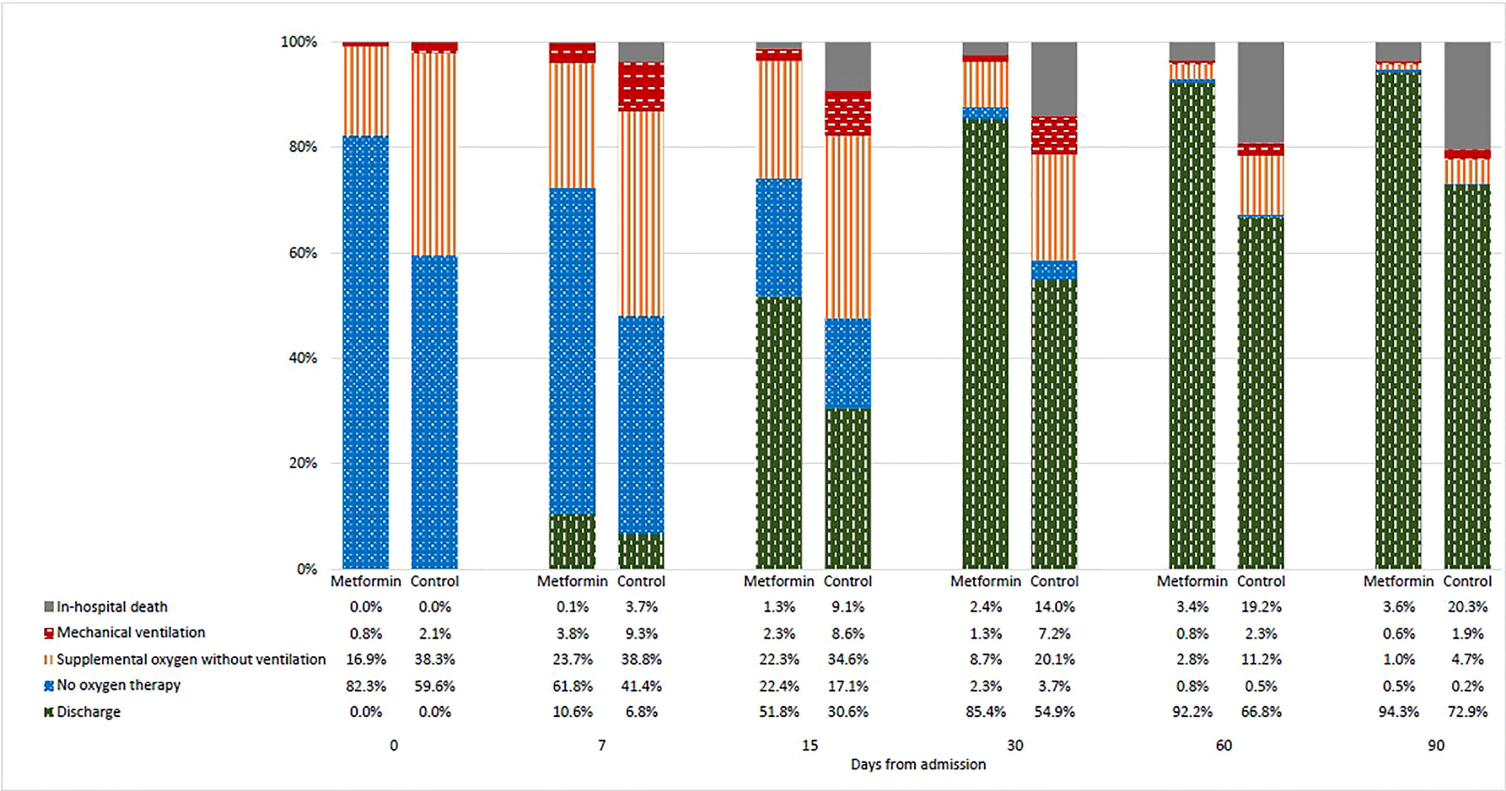
Changes in clinical status of patients as indicated by the WHO Clinical Progression Scale score from baseline to day-90 by treatment groups.

**Figure 2:**
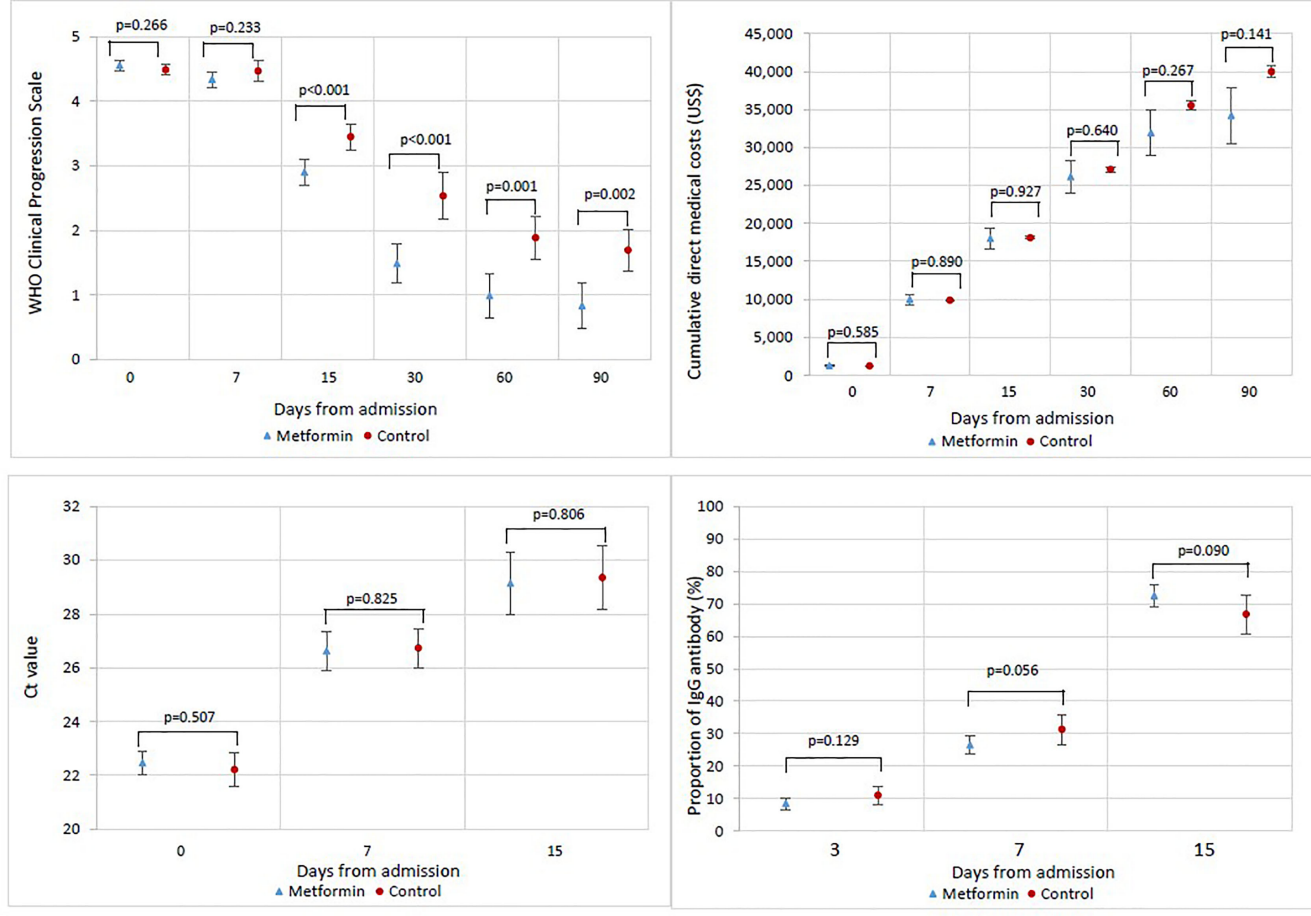
Changes in WHO Clinical Progression Scale score, cumulative direct medical costs, cycle threshold (Ct) value, and proportion of patients with IgG antibody from baseline to day-90 among patients by treatment groups.

Changes in the clinical status of patients over the follow-up period are illustrated in **Figure 1** by treatment group. As depicted in **Figure 2**, the mean WHO Clinical Progression Scale scores were significantly lower among metformin users compared with non-users since day 15 (2.91 vs. 3.45, *p* < 0.001; day 30: 1.49 vs. 2.53, *p* < 0.001; day 60: 0.99 vs. 1.88, *p* = 0.001; day 90: 0.83 vs. 1.69, *p* = 0.002). Both mean Ct values and the proportion of IgG antibody from baseline to day 15 yielded insignificant results between the exposure and non-exposure groups, despite a trend toward a higher proportion of patients with positive IgG antibodies in metformin users as demonstrated on day 15 (72% vs. 67%, *p* = 0.090). In terms of average cumulative direct medical costs incurred by treatment groups, metformin users had an insignificant increase in cost from baseline to day 7, followed by trends toward incurring insignificantly lower costs from day 7 to day 90 (US$34,199 vs. US$39,950, *p* = 0.141) compared with non-users.

Moreover, significantly increased likelihood of clinical improvement on the WHO Clinical Progression Scale by ≥1 score (HR: 1.27, 95% CI 1.03–1.58), increased risk of acidosis (HR: 8.48, 95% CI 1.48–48.57), and reduced risk of hepatic inflammation (HR: 0.59, 95% CI 0.39–0.89) can be seen in the additional analysis by time-to-event Cox models, which are consistent with the main analysis.

**Supplementary Table 5** summarizes the results of subgroup analyses. Significant results for the comparison between with and without preadmission use of metformin include higher odds to recovery, lower odds to macrophage activation, hepatic inflammation, and hyperinflammatory syndrome in the preadmission group. Without prior metformin use is associated with significantly lowered odds of mortality and increased odds to clinical improvement, hospital discharge, and acidosis.

## Discussion

It is important to review the risk–benefit balance of antidiabetic medication for T2DM patients amid the COVID-19 pandemic. Apart from metformin, drugs such as, but not limited to, DPP4i and SGLT2i have also been discussed. In-hospital DPP4i use was found to reduce the risk of mortality due to its potential anti-inflammatory effect proposed to be beneficial to T2DM COVID-19 patients while being generally well-tolerated with minimal side effects (18). On the other hand, SGLT2i was proposed to be used with greater precaution due to potentially higher risk of euglycemic diabetic ketoacidosis despite showing signs of reduction in production and expression of proinflammatory cytokines in both RCT and observational studies (19–21). Hence, it is of importance to help depict a clearer picture in the clinical treatment of COVID-19 in T2DM patients by understanding of the risks and benefits of metformin use.

In this retrospective, territory-wide cohort of T2DM patients hospitalized with COVID-19, metformin users were found to have significantly greater odds of clinical improvement, hospital discharge, and recovery, as well as a shorter length of hospital stay compared with their control counterparts. Despite significantly increased odds of acidosis, metformin use was associated with lower odds of mortality and hyperinflammatory syndrome compared with weighted control.

To the best of our knowledge, four meta-analyses have been conducted so far on the mortality outcome in metformin users with T2DM and COVID-19, which unanimously agreed that metformin use was associated with a significant reduction in mortality compared with control, and our results were generally in line with such observation (6, 22–24). Furthermore, the meta-analysis done by Yang et al. has demonstrated a significant reduction in disease severity among metformin users (24). Despite accumulating evidence pointing toward better clinical outcomes with reference to the anti-inflammatory effect of metformin and, hence, advocating for its use, several clinical guidelines and individual studies have suggested that it should be prescribed with caution due to its potential side effects of different severities, especially during the course of COVID-19; for instance, it should be withdrawn in cases of respiratory distress, renal impairment, and heart failure, owing to associated risks of lactic acidosis, ketoacidosis, and dehydration (4, 11, 25). Of note, Gao et al. echoed that metformin users might have significantly higher risks of disease progression and life-threatening complications compared with non-users (12).

It has been established that both T2DM and COVID-19 will contribute to a heightened inflammatory state in patients. While SARS-CoV-2 infection triggers acute immune responses, hyperglycemic state and poor blood glucose control in T2DM patients may lead to chronic inflammation characterized by innate immune system dysregulation (26, 27), ultimately increasing the risks of mortality and poor clinical outcomes (28–30). Therefore, the anti-inflammatory effect of metformin has been widely discussed as a viable option for T2DM patients with COVID-19, as users were found to have lower levels of proinflammatory cytokines and higher levels of anti-inflammatory cytokines compared with non-users (31), which may contribute to reducing the incidence of hyperinflammatory syndrome and, hence, widespread organ damage in these patients. To date, limited research has explored the relationship between metformin use and hyperinflammatory syndrome, while our results suggested a significant reduction in the odds of hyperinflammatory syndrome among metformin users when compared with control.

The currently proposed mechanistic explanations with regard to the anti-inflammatory effects of metformin remain disputed as metformin’s mechanism of action is closely tied to the expression and activity of ACE2, which has also been identified as a viral receptor for SARS-CoV-2 (25, 32). It is commonly acknowledged that metformin exerts glucose-lowering effects by activating the adenosine monophosphate-activated protein kinase (AMPK) (10). AMPK activates and phosphorylates ACE2 and, hence, evokes anti-inflammatory effects *via* catalyzing the production of angiotensin-(1-7) peptide of the RAS (33, 34). On the other hand, SARS-CoV-2 downregulates ACE2 expression upon cellular entry (8), which in turn promotes inflammation and potentially exacerbates hyperinflammatory syndrome, also known as cytokine storm, a key component of COVID-19 pathophysiology that is heavily implied in related mortality (35). The clash occurs with whether metformin, by potentially raising ACE2 expression with AMPK and Sirtuin 1 (25, 36), as well as increasing ACE2 stability by reducing ubiquitination and degradation (8, 10), would facilitate viral entry (12) and induce poor clinical outcomes, or would metformin be able to exert immunomodulatory effects to mitigate inflammation and organ damage. It has also been suggested that phosphorylated ACE2 after post-translational modification would be less recognizable by SARS-CoV-2 owing to steric hinderance (8, 10), thus shifting the action of ACE2 toward the cardiopulmonary protective alternative. Apart from acting on the RAS, AMPK activation is also associated with the reduction of nuclear factor kappa-light-chain-enhancer of activated B cells (NFκB) activity, lowering the release of proinflammatory cytokines and reducing mortality in women according to a recent retrospective cohort analysis (31). Our results are inconclusive regarding the notion of reduced viral entry *via* steric hindrance owing to insignificant differences in mean Ct values between groups; however, the lower odds of macrophage activation and hepatic inflammation, possibly induced by reduced cytokine release and signaling, immune cell activation, recruitment, and activity, seem to override the insignificant differences in viral clearance, hence leading to better clinical outcomes among metformin users.

Despite the positive result of lowering the odds of hyperinflammatory syndrome, our results also indicated significantly increased odds of acidosis in the treatment group compared with control, which has been consistently found in other studies (10, 12). Accordingly, the risk of acidosis might be a concern for specific patient subgroups (such as those with reduced kidney function and those with severe COVID-19). A mechanistic explanation proposed would be metformin’s inhibition of mitochondrial cellular respiration to enhance anaerobic respiration (37, 38), where the risk of acidosis may further be exacerbated owing to infection-induced hypoxia upon SARS-CoV-2 infection (39). Metformin use was associated with an increased risk of acidosis, but not mortality, as concluded by Cheng et al. (10), which was in line with the significantly reduced odds of mortality in our study. These results may imply that the protective effects of hyperinflammatory syndrome reduction could potentially override potential harms brought by acidosis. Nevertheless, the potential risk of lactic acidosis should still be meticulously acknowledged with careful monitoring of the patient’s condition and safe administration of metformin.

In this retrospective cohort study, all T2DM patients with COVID-19 were captured in the public healthcare system; hence, all eligible cases were included in this analysis regardless of disease severity. Besides, various patient characteristics at baseline were taken into account and balanced with multiple imputation and propensity score weighting, including pre-existing comorbidities and medical treatments of T2DM and COVID-19, as well as laboratory parameters on admission. While our study has provided some preliminary evidence on the association between metformin use and alleviation of hyperinflammatory syndrome in T2DM patients with COVID-19, our study was not without its limitations. Firstly, due to its observational nature, residual confounding might not have been fully addressed after propensity score weighting. Secondly, our patient cohort consisted of mainly Chinese and cases of moderate COVID-19, which will likely undermine the generalizability of our results to other populations or healthcare settings. Lastly, due to limited sample size of cases of “acidosis,” the breakdown of the “acidosis” outcome into “non-lactic acidosis” and “lactic acidosis” is not possible. It should be emphasized that although lactic acidosis is a potential adverse event of metformin therapy, it is extremely rare in the clinical setting.

In conclusion, metformin use was associated with significant increases in the odds of clinical improvement, hospital discharge, and recovery when compared with control, in addition to a shorter length of hospital stay. Despite an increased risk of acidosis, lower odds of in-hospital death and hyperinflammatory syndrome were observed among metformin users compared with their control counterparts. Our results demonstrated positive results regarding the management of inflammatory status and eventually clinical improvement of T2DM patients during COVID-19. Notably, as metformin treatment was associated with a significant risk of acidosis, patients with renal and/or pulmonary impairment should be carefully monitored. Prospective studies on the safe use of metformin are required for better clinical management of T2DM patients with COVID-19.

## Data Availability Statement

The data that supported the findings of this study were provided by the Hong Kong Hospital Authority but restrictions apply to the availability of these data, which were used under license for the current study, and so are not publicly available. Data are however available from the authors upon reasonable request and with permission of Hong Kong Hospital Authority.

## Ethics Statement

The study protocol was approved by the Institutional Review Board of the University of Hong Kong/ Hospital Authority Hong Kong West Cluster (Reference No. UW 20-493). Given the extraordinary nature of the COVID-19 pandemic, individual patient informed consent was not required for this retrospective cohort study using anonymized data. 

## Author Contributions

CW reviewed the literature, designed the statistical analysis, conducted the analyses, and wrote the manuscript. DL, AL, AK, ML, and KL reviewed the literature, contributed to the interpretation and analysis of data, and wrote the manuscript. IA and MC conducted the analyses. XX, EL, and BC contributed to the interpretation and analysis of data. All authors contributed to the interpretation and analysis of data, critically reviewed and revised the manuscript, and approved the final manuscript as submitted. The corresponding author attests that all listed authors meet authorship criteria and that no others meeting the criteria have been omitted.

## Funding

We received financial support from the Health and Medical Research Fund, Food and Health Bureau, Government of the Hong Kong Special Administrative Region, China (grant no. COVID190210). The funders did not have any role in the design and conduct of the study; collection, management, analysis, and interpretation of the data; preparation, review, or approval of the manuscript; and decision to submit the manuscript for publication.

## Conflict of Interest

The authors declare that the research was conducted in the absence of any commercial or financial relationships that could be construed as a potential conflict of interest.

## Publisher’s Note

All claims expressed in this article are solely those of the authors and do not necessarily represent those of their affiliated organizations, or those of the publisher, the editors and the reviewers. Any product that may be evaluated in this article, or claim that may be made by its manufacturer, is not guaranteed or endorsed by the publisher.

## References

[1] Who Coronavirus (Covid-19) Dashboard. Available at: https://covid19.who.int/ (Accessed 19/08, 2021).

[2] Barron E Bakhai C Kar P Weaver A Bradley D Ismail H. Associations of Type 1 and Type 2 Diabetes With COVID-19-Related Mortality in England: A Whole-Population Study. Lancet Diabetes Endocrinol (2020) 8(10):813–22. doi: 10.1016/S2213-8587(20)30272-2 PMC7426088 32798472

[3] Sardu C D’Onofrio N Balestrieri ML Barbieri M Rizzo MR Messina V. Outcomes in Patients With Hyperglycemia Affected by COVID-19: Can We do More on Glycemic Control? Diabetes Care (2020) 43(7):1408–15. doi: 10.2337/dc20-0723 PMC7305003 32430456

[4] Bornstein SR Rubino F Khunti K Mingrone G Hopkins D Birkenfeld AL. Practical Recommendations for the Management of Diabetes in Patients With COVID-19. Lancet Diabetes Endocrinol (2020) 8(6):546–50. doi: 10.1016/S2213-8587(20)30152-2 PMC7180013 32334646

[5] Papazafiropoulou AK Antonopoulos S. The COVID-19 Pandemic and Diabetes Mellitus. Arch Med Sci Atherosclerotic Dis (2020) 5:e200. doi: 10.5114/amsad.2020.97435 PMC7433782 32832721

[6] Lukito AA Pranata R Henrina J Lim MA Lawrensia S Suastika K. The Effect of Metformin Consumption on Mortality in Hospitalized COVID-19 Patients: A Systematic Review and Meta-Analysis. Diabetes Metab Syndrome: Clin Res Rev (2020) 14(6):2177–83. doi: 10.1016/j.dsx.2020.11.006 PMC7657016 33395778

[7] Zhu L She Z-G Cheng X Qin J-J Zhang X-J Cai J. Association of Blood Glucose Control and Outcomes in Patients With COVID-19 and Pre-Existing Type 2 Diabetes. Cell Metab (2020) 31(6):1068–77.e3. doi: 10.1016/j.cmet.2020.04.021 32369736 PMC7252168

[8] Sharma S Ray A Sadasivam B. Metformin in COVID-19: A Possible Role Beyond Diabetes. Diabetes Res Clin Practice (2020) 164:108183. doi: 10.1016/j.diabres.2020.108183 PMC7190487 32360697

[9] Luo P Qiu L Liu Y Liu X-L Zheng J-L Xue H-Y. Metformin Treatment was Associated With Decreased Mortality in COVID-19 Patients With Diabetes in a Retrospective Analysis. Am J Trop Med Hygiene (2020) 103(1):69. doi: 10.4269/ajtmh.20-0375 PMC7356425 32446312

[10] Cheng X Liu Y-M Li H Zhang X Lei F Qin J-J. Metformin Is Associated With Higher Incidence of Acidosis, But Not Mortality, in Individuals With COVID-19 and Pre-Existing Type 2 Diabetes. Cell Metab (2020) 32(4):537–47.e3. doi: 10.1016/j.cmet.2020.08.013 32861268 PMC7439986

[11] Apicella M Campopiano MC Mantuano M Mazoni L Coppelli A Del Prato S. COVID-19 in People With Diabetes: Understanding the Reasons for Worse Outcomes. Lancet Diabetes Endocrinol (2020) 8(9):782–92. doi: 10.1016/S2213-8587(20)30238-2 PMC7367664 32687793

[12] Gao Y Liu T Zhong W Liu R Zhou H Huang W. Risk of Metformin in Patients With Type 2 Diabetes With COVID-19: A Preliminary Retrospective Report. Clin Trans Sci (2020) 13(6):1055–9. doi: 10.1111/cts.12897 PMC7537216 32955785

[13] Concise Advice on Inpatient Diabetes (COVID: Diabetes): Hyperglycaemia/Diabetes Guidance for People With Covid-19 Infections Managed in a Virtual Ward: a Guide for Healthcare Professionals The Association of British Clinical Diabetologists (ABCD) (2021).

[14] Wong CKH Lui DTW Lui AYC Kwok ACY Low MCH Lau KTK. Use of DPP4i Reduced Odds of Clinical Deterioration and Hyperinflammatory Syndrome in COVID-19 Patients With Type 2 Diabetes: Propensity Score Analysis of a Territory-Wide Cohort in Hong Kong. Diabetes Metab (2022) 48(1):101307. doi: 10.1016/j.diabet.2021.101307 34863934 PMC8632053

[15] WHO Working Group on the Clinical Characterisation and Management of COVID-19 infection. A Minimal Common Outcome Measure Set for COVID-19 Clinical Research. Lancet Infect Dis (2020) 20(8):e192–7. doi: 10.1016/s1473-3099(20)30483-7 PMC7292605 32539990

[16] Webb BJ Peltan ID Jensen P Hoda D Hunter B Silver A. Clinical Criteria for COVID-19-Associated Hyperinflammatory Syndrome: A Cohort Study. Lancet Rheumatol (2020) 2(12):e754–63. doi: 10.1016/S2665-9913(20)30343-X PMC7524533 33015645

[17] Tom MR Mina MJ. To Interpret the SARS-CoV-2 Test, Consider the Cycle Threshold Value. Clin Infect Dis (2020) 71(6):2252–54. doi: 10.1093/cid/ciaa619 PMC7314112 32435816

[18] Pal R Banerjee M Mukherjee S Bhogal RS Kaur A Bhadada SK. Dipeptidyl Peptidase-4 Inhibitor Use and Mortality in COVID-19 Patients With Diabetes Mellitus: An Updated Systematic Review and Meta-Analysis. Ther Adv Endocrinol Metab (2021) 12:2042018821996482. doi: 10.1177/2042018821996482 33680425 PMC7897812

[19] Koufakis T Maltese G Metallidis S Zebekakis P Kotsa K. Looking Deeper Into the Findings of DARE-19: Failure or an Open Door to Future Success? Pharmacol Res (2021) 173:105872. doi: 10.1016/j.phrs.2021.105872 34487851 PMC8416358

[20] Koufakis T Pavlidis AN Metallidis S Kotsa K. Sodium-Glucose Co-Transporter 2 Inhibitors in COVID-19: Meeting at the Crossroads Between Heart, Diabetes and Infectious Diseases. Int J Clin Pharmacy (2021) 1–4. doi: 10.1007/s11096-021-01256-9 PMC7942520 33751323

[21] Koufakis T Metallidis S Zebekakis P Ajjan RA Kotsa K. Sodium-Glucose Cotransporter 2 Inhibitors in the Era of COVID-19 Pandemic: Is the Benefit to Risk Ratio Still Favorable? J Diabetes Sci Tech (2020) 14(4):745–7. doi: 10.1177/1932296820932155 PMC7673172 32486846

[22] Hariyanto TI Kurniawan A. Metformin Use Is Associated With Reduced Mortality Rate From Coronavirus Disease 2019 (COVID-19) Infection. Obes Med (2020) 19:100290. doi: 10.1016/j.obmed.2020.100290 32844132 PMC7434427

[23] Kow CS Hasan SS. Mortality Risk With Preadmission Metformin Use in Patients With COVID-19 and Diabetes: A Meta-Analysis. J Med Virol (2021) 93(2):695–7. doi: 10.1002/jmv.26498 32902868

[24] Yang W Sun X Zhang J Zhang K. The Effect of Metformin on Mortality and Severity in COVID-19 Patients With Diabetes Mellitus. Diabetes Res Clin Practice (2021) 178:108977. doi: 10.1016/j.diabres.2021.108977 PMC8295100 34302912

[25] Sharif-Askari NS Sharif-Askari FS Mdkhana B Al Heialy S Ratemi E Alghamdi M. Effect of Common Medications on the Expression of SARS-CoV-2 Entry Receptors in Liver Tissue. Arch Toxicol (2020) 94(12):4037–41. doi: 10.1007/s00204-020-02869-1 PMC7430937 32808185

[26] Ilyas R Wallis R Soilleux EJ Townsend P Zehnder D Tan BK. High Glucose Disrupts Oligosaccharide Recognition Function via Competitive Inhibition: A Potential Mechanism for Immune Dysregulation in Diabetes Mellitus. Immunobiology (2011) 216(1-2):126–31. doi: 10.1016/j.imbio.2010.06.002 PMC3088832 20674073

[27] Bode B Garrett V Messler J McFarland R Crowe J Booth R. Glycemic Characteristics and Clinical Outcomes of COVID-19 Patients Hospitalized in the United States. J Diabetes Sci Tech (2020) 14(4):813–21. doi: 10.1177/1932296820924469 PMC7673150 32389027

[28] Mirabelli M Chiefari E Puccio L Foti DP Brunetti A. Potential Benefits and Harms of Novel Antidiabetic Drugs During COVID-19 Crisis. Int J Environ Res Public Health (2020) 17(10):3664. doi: 10.3390/ijerph17103664 PMC7277613 32456064

[29] Chen Y Yang D Cheng B Chen J Peng A Yang C. Clinical Characteristics and Outcomes of Patients With Diabetes and COVID-19 in Association With Glucose-Lowering Medication. Diabetes Care (2020) 43(7):1399–407. doi: 10.2337/dc20-0660 32409498

[30] Riahi S Sombra LRS Lo KB Chacko SR Neto AGM Azmaiparashvili Z. Insulin Use, Diabetes Control, and Outcomes in Patients With COVID-19. Endocrine Res (2021) 46(2):45–50. doi: 10.1080/07435800.2020.1856865 33275067

[31] Bramante CT Ingraham NE Murray TA Marmor S Hovertsen S Gronski J. Metformin and Risk of Mortality in Patients Hospitalised With COVID-19: A Retrospective Cohort Analysis. Lancet Healthy Longevity (2021) 2(1):e34–41. doi: 10.1016/S2666-7568(20)30033-7 PMC7832552 33521772

[32] Xiao L Sakagami H Miwa N. ACE2: The Key Molecule for Understanding the Pathophysiology of Severe and Critical Conditions of COVID-19: Demon or Angel? Viruses (2020) 12(5):491. doi: 10.3390/v12050491 PMC7290508 32354022

[33] Katulanda P Dissanayake HA Ranathunga I Ratnasamy V Wijewickrama PSA Yogendranathan N. Prevention and Management of COVID-19 Among Patients With Diabetes: An Appraisal of the Literature. Diabetologia (2020) 63(8):1440–52. doi: 10.1007/s00125-020-05164-x PMC7220850 32405783

[34] Lumbers ER Delforce SJ Pringle KG Smith GR. The Lung, the Heart, the Novel Coronavirus, and the Renin-Angiotensin System; the Need for Clinical Trials. Front Med (2020) 7:248. doi: 10.3389/fmed.2020.00248 PMC7256451 32574336

[35] Hu B Huang S Yin L. The Cytokine Storm and COVID-19. J Med Virol (2021) 93(1):250–6. doi: 10.1002/jmv.26232 PMC7361342 32592501

[36] Zhang J Dong J Martin M He M Gongol B Marin TL. AMP-Activated Protein Kinase Phosphorylation of Angiotensin-Converting Enzyme 2 in Endothelium Mitigates Pulmonary Hypertension. Am J Respir Crit Care Med (2018) 198(4):509–20. doi: 10.1164/rccm.201712-2570OC PMC6118028 29570986

[37] Lalau J-D Arnouts P Sharif A De Broe ME. Metformin and Other Antidiabetic Agents in Renal Failure Patients. Kidney Int (2015) 87(2):308–22. doi: 10.1038/ki.2014.19 24599253

[38] Kopec KT Kowalski MJ. Metformin-Associated Lactic Acidosis (MALA): Case Files of the Einstein Medical Center Medical Toxicology Fellowship. J?Med Toxicol (2013) 9(1):61–6. doi: 10.1007/s13181-012-0278-3 PMC3576503 23233435

[39] Suetrong B Walley KR. Lactic Acidosis in Sepsis: It’s Not All Anaerobic: Implications for Diagnosis and Management. Chest (2016) 149(1):252–61. doi: 10.1378/chest.15-1703 26378980

