# Supplementary material for: Metformin Use in Relation to Clinical Outcomes and Hyperinflammatory Syndrome Among COVID-19 Patients With Type 2 Diabetes: A Propensity Score Analysis of a Territory-Wide Cohort
Source: Front Endocrinol (Lausanne). 2022 Mar 7;13:810914. doi: 10.3389/fendo.2022.810914 (PMC8935075; doi:10.3389/fendo.2022.810914)
Supplement: Supplementary file 1 [file DataSheet_1.pdf]

Supplementary Figure 1. Distribution of propensity score density of metformin and control groups before weighting (a) and after weighting (b)

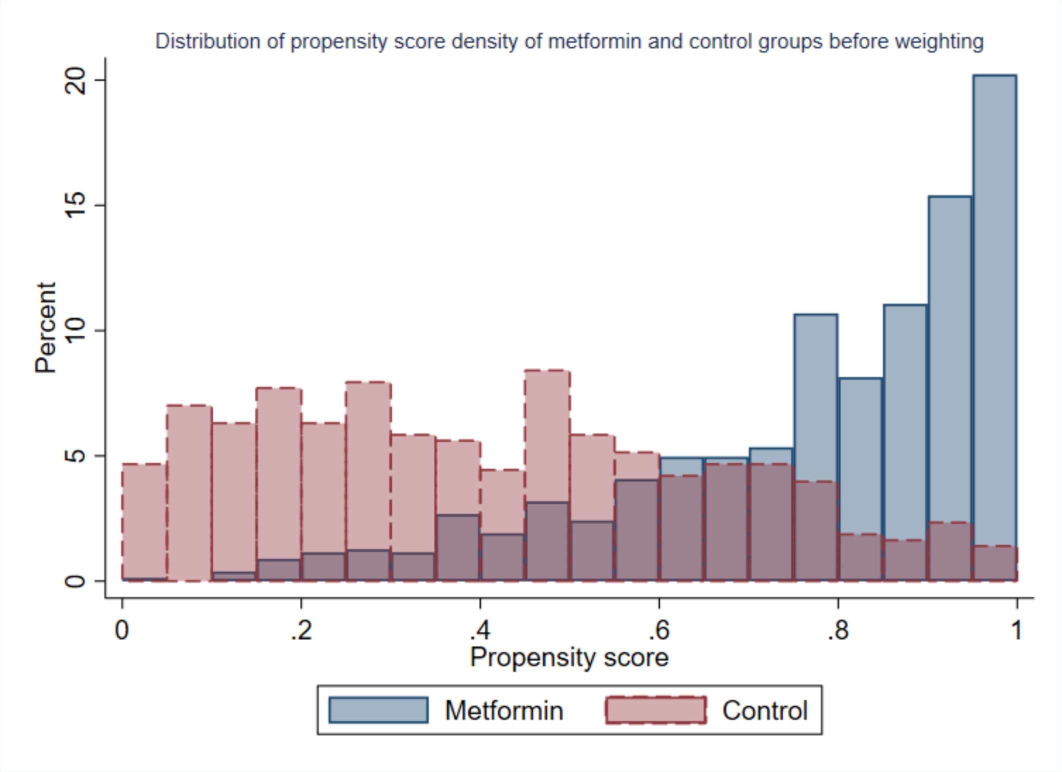

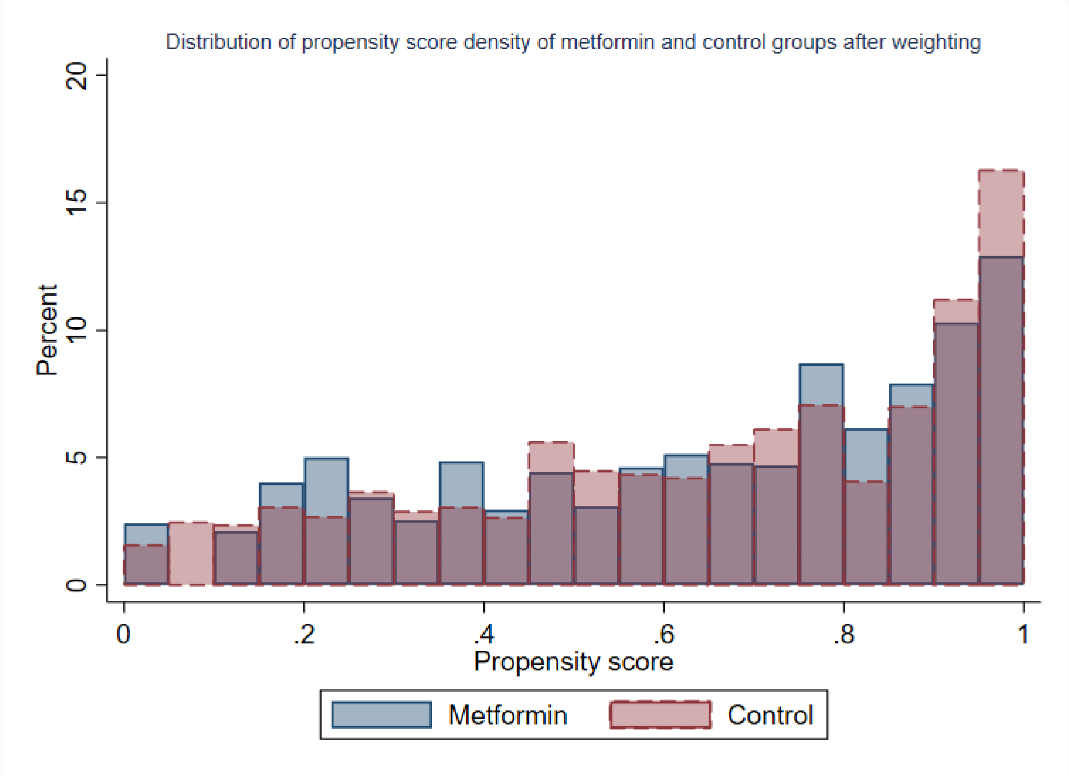

Supplementary Table 1. Unit cost of healthcare services

Health Service	Cost (HK$)	Cost (US$)†
Hospitalization, per day		
General Ward	5,100	654
Intensive Care Unit	24,400	3,128
Emergency department, per visit	1,230	158
Polymerase chain reaction tests	240	31
Extracorporeal membrane oxygenation (ECMO) catheter insertion	52,100	6,679
ECMO	80,175	10,279
Tracheostomy	16,050	2,058
Hemodialysis	9,410	1,206
Peritoneal dialysis	9,410	1,206

Data source: Hong Kong SAR Government Gazette and Hospital Authority Ordinance (chapter 113): Public charges – non-eligible persons.

Note: ECMO = Extracorporeal membrane oxygenation

†A fixed exchange rate of 1 US$=7.8 HK$

Supplementary Table 2. Data completion rates of baseline characteristics before multiple imputation

Characteristics	Total (N = 1,214)	Metformin (N=786)	Control (N=428)
Age	100.0% (1,214)	100.0% (786)	100.0% (428)
Sex	100.0% (1,214)	100.0% (786)	100.0% (428)
Pre-existing comorbidities on admission			
Charlson's Index†	100.0% (1,214)	100.0% (786)	100.0% (428)
Diabetes mellitus	100.0% (1,214)	100.0% (786)	100.0% (428)
Hypertension	100.0% (1,214)	100.0% (786)	100.0% (428)
Chronic heart disease	100.0% (1,214)	100.0% (786)	100.0% (428)
Chronic lung disease	100.0% (1,214)	100.0% (786)	100.0% (428)
Chronic kidney disease	100.0% (1,214)	100.0% (786)	100.0% (428)
Liver disease	100.0% (1,214)	100.0% (786)	100.0% (428)
Malignancy	100.0% (1,214)	100.0% (786)	100.0% (428)
Obesity	100.0% (1,214)	100.0% (786)	100.0% (428)
Preadmission or in-hospital use			
Metformin	100.0% (1,214)	100.0% (786)	100.0% (428)
GLP1RA	100.0% (1,214)	100.0% (786)	100.0% (428)
Insulin	100.0% (1,214)	100.0% (786)	100.0% (428)
Oral anti-diabetic drugs			
SU	100.0% (1,214)	100.0% (786)	100.0% (428)
TZD	100.0% (1,214)	100.0% (786)	100.0% (428)
Acarbose	100.0% (1,214)	100.0% (786)	100.0% (428)
DPP4i	100.0% (1,214)	100.0% (786)	100.0% (428)
SGLT2i	100.0% (1,214)	100.0% (786)	100.0% (428)
Long-term medications			
ACEI or ARB	100.0% (1,214)	100.0% (786)	100.0% (428)
Anticoagulant	100.0% (1,214)	100.0% (786)	100.0% (428)
Antiplatelet	100.0% (1,214)	100.0% (786)	100.0% (428)
Lipid-lowering agent	100.0% (1,214)	100.0% (786)	100.0% (428)
NSAID	100.0% (1,214)	100.0% (786)	100.0% (428)
In-hospital COVID-19 drug use			
Remdesivir	100.0% (1,214)	100.0% (786)	100.0% (428)
Interferon-β-1b	100.0% (1,214)	100.0% (786)	100.0% (428)
Dexamethasone	100.0% (1,214)	100.0% (786)	100.0% (428)
Tocilizumab	100.0% (1,214)	100.0% (786)	100.0% (428)
ECMO	100.0% (1,214)	100.0% (786)	100.0% (428)
Dialysis	100.0% (1,214)	100.0% (786)	100.0% (428)
ICU admission on admission	100.0% (1,214)	100.0% (786)	100.0% (428)
Admission via emergency department	100.0% (1,214)	100.0% (786)	100.0% (428)

Clinical severity on admission§	100.0% (1,214)	100.0% (786)	100.0% (428)
ARDS	100.0% (1,214)	100.0% (786)	100.0% (428)
Macrophage activation	100.0% (1,214)	100.0% (786)	100.0% (428)
Haematological dysfunction	100.0% (1,214)	100.0% (786)	100.0% (428)
Coagulopathy	100.0% (1,214)	100.0% (786)	100.0% (428)
Hepatic inflammation	100.0% (1,214)	100.0% (786)	100.0% (428)
Hyperinflammatory syndrome	100.0% (1,214)	100.0% (786)	100.0% (428)
Laboratory parameters on admission			
White blood cell	99.9% (1,213)	100.0% (786)	99.8% (427)
Neutrophil	99.4% (1,207)	99.6% (783)	99.1% (424)
Lymphocyte	99.4% (1,207)	99.6% (783)	99.1% (424)
Platelet	99.8% (1,212)	100.0% (786)	99.5% (426)
Lactate dehydrogenase	97.4% (1,183)	97.7% (768)	97.0% (415)
Creatine kinase	90.8% (1,102)	89.8% (706)	92.5% (396)
Total bilirubin	100.0% (1,214)	100.0% (786)	100.0% (428)
C-reactive protein	97.5% (1,184)	97.5% (766)	97.7% (418)
Cycle threshold value	92.4% (1,122)	92.9% (730)	91.6% (392)
eGFR	100.0% (1,214)	100.0% (786)	100.0% (428)
ALP	98.1% (1,191)	97.8% (769)	98.6% (422)
ALT	100.0% (1,214)	100.0% (786)	100.0% (428)
Hemoglobin	99.9% (1,213)	100.0% (786)	99.8% (427)
Prothrombin time	43.8% (532)	44.0% (346)	43.5% (186)
Random glucose	53.3% (647)	54.8% (431)	50.5% (216)
Hemoglobin A1c	27.2% (330)	31.8% (250)	18.7% (80)

Note: ACEI = Angiotensin converting enzyme inhibitor; ALP = Alkaline phosphatase; ALT = Alanine transaminase; ARB = Angiotensin receptor blockers; AST = Aspartate aminotransferase; ECMO = Extracorporeal membrane oxygenation; eGFR= Estimated glomerular filtration rate; ICU = Intensive Care Unit; NSAID = Nonsteroidal anti-inflammatory drugs

† The calculation of Charlson Index does not include Acquired Immune Deficiency Syndrome (AIDS).

§ Clinical severity is classified according to WHO Clinical Progression Scale

Supplementary Table 3. Number and incidence rate of outcome events

Event	Before weighting							After weighting	
	Cumulative incidence		Crude incidence rate (Events / 100 person-days)			Median follow-up periods (Days)	Mean follow-up periods (Days)	Incidence rate (Events / 100 person-days)	
	Cases with event	Rate	Estimate	95% CI*	Person-days			Estimate	95% CI*
**Total (N = 1214)**									
Clinical improvement on WHO clinical progression scale by ≥ 1 score	1107	0.9119	3.943	(3.714, 4.183)	28073	15	23	4.028	(3.866, 4.194)
Clinical improvement on WHO clinical progression scale by ≥ 2 scores	1097	0.9036	3.706	(3.490, 3.932)	29604	16	24	3.720	(3.570, 3.874)
Hospital discharge (score ≤ 3)	1080	0.8896	3.596	(3.385, 3.817)	30035	16	25	3.547	(3.403, 3.695)
Recovery (score ≤ 4)	223	0.7147	1.566	(1.367, 1.786)	14237	30	46	1.630	(1.486, 1.783)
Viral clearance (first negative PCR result)	1024	0.9615	10.465	(9.834, 11.126)	9785	8	9	10.621	(10.179, 11.075)
Low viral load (Ct value ≥ 35)	386	0.3268	1.809	(1.633, 1.999)	21340	14	18	1.967	(1.839, 2.102)
Low viral load (Ct value ≥ 30)	908	0.8277	7.085	(6.632, 7.561)	12816	10	12	7.642	(7.310, 7.985)
Seroconversion (first Ct value ≥ 30 or IgG antibody positivity)	1106	0.9110	7.576	(7.136, 8.036)	14599	11	13	7.952	(7.636, 8.277)
IgG antibody	1045	0.8608	6.674	(6.276, 7.092)	15657	11	13	6.755	(6.477, 7.040)
In-hospital death or invasive mechanical ventilation (score ≥ 7)	239	0.1993	1.140	(1.000, 1.294)	20965	14	17	1.095	(1.001, 1.195)
In-hospital death or invasive mechanical ventilation (score ≥ 7) or intensive care unit admission	90	0.0938	0.571	(0.459, 0.702)	15765	14	16	0.648	(0.568, 0.737)
In-hospital death, invasive mechanical ventilation, vasopressors, dialysis, or ECMO (score ≥ 9)	141	0.1165	0.483	(0.406, 0.569)	29205	16	24	0.508	(0.453, 0.566)
In-hospital death (score = 10)	115	0.0947	0.383	(0.316, 0.460)	30035	16	25	0.410	(0.362, 0.462)
Acidosis	13	0.0107	0.044	(0.023, 0.075)	29767	16	25	0.084	(0.063, 0.109)
Hypoglycemia	45	0.0371	0.151	(0.110, 0.203)	29710	16	25	0.088	(0.066, 0.114)
ARDS	130	0.1097	0.556	(0.464, 0.660)	23397	14	20	0.506	(0.446, 0.571)
Macrophage activation	432	0.3558	2.571	(2.334, 2.825)	16804	10	14	2.628	(2.463, 2.799)
Haematological dysfunction	412	0.3636	3.115	(2.822, 3.431)	13226	10	12	2.892	(2.698, 3.092)
Coagulopathy	115	0.0952	0.471	(0.389, 0.565)	24429	15	20	0.363	(0.313, 0.417)
Hepatic inflammation	351	0.3131	2.333	(2.095, 2.590)	15046	11	13	2.349	(2.186, 2.517)
Hyperinflammatory syndrome	571	0.5397	5.853	(5.383, 6.353)	9756	7	9	5.803	(5.485, 6.130)
**Metformin (N = 786)**									

Clinical improvement on WHO clinical progression scale by ≥ 1 score	763	0.9707	4.850	(4.512, 5.207)	15732	15	20	4.531	(4.277, 4.794)
Clinical improvement on WHO clinical progression scale by ≥ 2 scores	758	0.9644	4.674	(4.347, 5.019)	16218	15	21	4.150	(3.917, 4.394)
Hospital discharge (score ≤ 3)	752	0.9567	4.594	(4.272, 4.934)	16369	15	21	3.855	(3.635, 4.080)
Recovery (score ≤ 4)	119	0.8561	2.164	(1.792, 2.589)	5500	27	40	1.861	(1.643, 2.094)
Viral clearance (first negative PCR result)	681	0.9927	11.480	(10.634, 12.376)	5932	8	9	11.020	(10.365, 11.702)
Low viral load (Ct value ≥ 35)	238	0.3115	1.935	(1.697, 2.197)	12301	13	16	1.938	(1.753, 2.135)
Low viral load (Ct value ≥ 30)	601	0.8513	7.766	(7.157, 8.412)	7739	9	11	7.440	(6.974, 7.921)
Seroconversion (first Ct value ≥ 30 or IgG antibody positivity)	738	0.9389	8.137	(7.560, 8.746)	9070	11	13	7.805	(7.363, 8.263)
IgG antibody	702	0.8931	7.242	(6.716, 7.798)	9694	10	12	7.074	(6.665, 7.500)
In-hospital death or invasive mechanical ventilation (score ≥ 7)	80	0.1026	0.604	(0.479, 0.752)	13238	14	17	0.886	(0.762, 1.020)
In-hospital death or invasive mechanical ventilation (score ≥ 7) or intensive care unit admission	33	0.0484	0.296	(0.204, 0.416)	11148	14	16	0.443	(0.347, 0.554)
In-hospital death, invasive mechanical ventilation, vasopressors, dialysis, or ECMO (score ≥ 9)	39	0.0497	0.245	(0.175, 0.336)	15886	15	20	0.334	(0.271, 0.407)
In-hospital death (score = 10)	28	0.0356	0.171	(0.114, 0.247)	16369	15	21	0.264	(0.209, 0.328)
Acidosis	9	0.0115	0.056	(0.025, 0.106)	16189	15	21	0.160	(0.117, 0.213)
Hypoglycemia	35	0.0446	0.217	(0.151, 0.301)	16148	15	21	0.131	(0.093, 0.179)
ARDS	57	0.0737	0.413	(0.313, 0.535)	13808	14	18	0.553	(0.462, 0.654)
Macrophage activation	239	0.3041	2.257	(1.980, 2.562)	10589	11	13	2.505	(2.271, 2.753)
Haematological dysfunction	205	0.2752	2.228	(1.933, 2.555)	9201	11	12	2.791	(2.514, 3.087)
Coagulopathy	48	0.0613	0.341	(0.251, 0.452)	14089	14	18	0.358	(0.287, 0.440)
Hepatic inflammation	170	0.2304	1.622	(1.388, 1.886)	10478	12	14	1.641	(1.457, 1.838)
Hyperinflammatory syndrome	344	0.4879	4.998	(4.484, 5.555)	6883	8	10	5.591	(5.137, 6.074)
**Control (N = 428)**									
Clinical improvement on WHO clinical progression scale by ≥ 1 score	344	0.8037	2.787	(2.501, 3.098)	12341	18	29	3.612	(3.406, 3.825)
Clinical improvement on WHO clinical progression scale by ≥ 2 scores	339	0.7921	2.532	(2.270, 2.817)	13386	18	31	3.361	(3.168, 3.560)
Hospital discharge (score ≤ 3)	328	0.7664	2.400	(2.147, 2.674)	13666	19	32	3.275	(3.087, 3.472)
Recovery (score ≤ 4)	104	0.6012	1.190	(0.973, 1.442)	8737	35	51	1.401	(1.214, 1.604)
Viral clearance (first negative PCR result)	343	0.9050	8.902	(7.985, 9.896)	3853	9	10	10.272	(9.680, 10.889)
Low viral load (Ct value ≥ 35)	148	0.3549	1.637	(1.384, 1.923)	9039	16	22	1.994	(1.816, 2.182)
Low viral load (Ct value ≥ 30)	307	0.7852	6.047	(5.389, 6.763)	5077	12	13	7.842	(7.370, 8.335)

Seroconversion (first Ct value ≥ 30 or IgG antibody positivity)	368	0.8598	6.656	(5.993, 7.372)	5529	12	14	8.099	(7.649, 8.563)
IgG antibody	343	0.8014	5.752	(5.159, 6.394)	5963	11	14	6.464	(6.090, 6.853)
In-hospital death or invasive mechanical ventilation (score ≥ 7)	159	0.3795	2.058	(1.750, 2.404)	7727	14	18	1.278	(1.138, 1.426)
In-hospital death or invasive mechanical ventilation (score ≥ 7) or intensive care unit admission	57	0.2050	1.235	(0.935, 1.600)	4617	13	17	0.829	(0.703, 0.966)
In-hospital death, invasive mechanical ventilation, vasopressors, dialysis, or ECMO (score ≥ 9)	102	0.2400	0.766	(0.624, 0.930)	13319	18	31	0.660	(0.574, 0.751)
In-hospital death (score = 10)	87	0.2033	0.637	(0.510, 0.785)	13666	19	32	0.540	(0.464, 0.622)
Acidosis	4	0.0093	0.029	(0.008, 0.075)	13578	19	32	0.020	(0.007, 0.040)
Hypoglycemia	10	0.0234	0.074	(0.035, 0.136)	13562	19	32	0.050	(0.029, 0.079)
ARDS	73	0.1772	0.761	(0.597, 0.957)	9589	15	23	0.465	(0.387, 0.552)
Macrophage activation	193	0.4509	3.105	(2.683, 3.576)	6215	9	15	2.736	(2.506, 2.977)
Haematological dysfunction	207	0.5335	5.143	(4.466, 5.893)	4025	8	10	2.977	(2.712, 3.256)
Coagulopathy	67	0.1576	0.648	(0.502, 0.823)	10340	16	24	0.367	(0.301, 0.443)
Hepatic inflammation	181	0.4726	3.962	(3.406, 4.584)	4568	9	12	3.148	(2.877, 3.437)
Hyperinflammatory syndrome	227	0.6431	7.901	(6.907, 8.999)	2873	6	8	5.983	(5.546, 6.440)

Note: ARDS = Acute respiratory distress syndrome; CI = confidence interval; Ct = cycle threshold; IgG = Immunoglobulin G; PCR = polymerase chain reaction

* 95% CI of incidence rates were constructed by Poisson distribution.

Supplementary Table 4. Sensitivity analysis on the outcomes

	Before weighting		After weighting		
	Metformin	Control	Metformin vs Control		
	% (N)	% (N)	OR†	95% CI	P-value
**Clinical improvement on WHO clinical progression scale by ≥ 1 score**					
Removing hospital discharge as censoring criteria	97.1% (786)	80.4% (428)	2.74	(1.31, 5.71)	0.009
At most 90 days of follow-up period	97.1% (786)	80.4% (428)	2.74	(1.31, 5.71)	0.009
**Hospital discharge (score ≤ 3)**					
Removing hospital discharge as censoring criteria	95.7% (786)	76.6% (428)	2.26	(1.24, 4.12)	0.009
At most 90 days of follow-up period	95.7% (786)	76.6% (428)	2.26	(1.24, 4.12)	0.009
**Recovery (score ≤ 4)**					
Removing hospital discharge as censoring criteria	85.6% (139)	60.1% (173)	2.54	(1.34, 4.80)	0.005
At most 90 days of follow-up period	85.6% (139)	60.1% (173)	2.54	(1.34, 4.80)	0.005
**In-hospital death (score = 10)**					
Removing hospital discharge as censoring criteria	1.1% (786)	0.9% (428)	0.64	(0.43, 0.96)	0.031
At most 90 days of follow-up period	1.1% (786)	0.9% (428)	0.64	(0.43, 0.95)	0.028
**Acidosis**					
Removing hospital discharge as censoring criteria	4.5% (784)	2.3% (428)	6.82	(2.56, 18.18)	<0.001
At most 90 days of follow-up period	4.5% (784)	2.3% (428)	6.82	(2.56, 18.18)	<0.001
**Macrophage activation**					
Removing hospital discharge as censoring criteria	27.5% (745)	53.4% (388)	0.74	(0.59, 0.93)	0.009
At most 90 days of follow-up period	27.5% (745)	53.4% (388)	0.74	(0.59, 0.93)	0.009
**Haematological dysfunction**					
Removing hospital discharge as censoring criteria	6.1% (783)	15.8% (425)	0.77	(0.55, 1.10)	0.142
At most 90 days of follow-up period	6.1% (783)	15.8% (425)	0.77	(0.55, 1.10)	0.142
**Coagulopathy**					
Removing hospital discharge as censoring criteria	23.0% (738)	47.3% (383)	0.81	(0.58, 1.14)	0.234
At most 90 days of follow-up period	23.0% (738)	47.3% (383)	0.81	(0.58, 1.14)	0.234
**Hepatic inflammation**					
Removing hospital discharge as censoring criteria	48.8% (705)	64.3% (353)	0.50	(0.38, 0.65)	<0.001
At most 90 days of follow-up period	48.8% (705)	64.3% (353)	0.50	(0.38, 0.65)	<0.001
**Hyperinflammatory syndrome**					
Removing hospital discharge as censoring criteria	19.2% (682)	32.7% (278)	0.71	(0.53, 0.95)	0.021
At most 90 days of follow-up period	19.1% (682)	32.7% (278)	0.71	(0.53, 0.95)	0.021

Note: CI = confidence interval; Ct = cycle threshold; OR = odds ratio; IgG = Immunoglobulin G; IPTW= Inverse probability of treatment weighting

† OR >1 (or <1) indicates metformin group was associated with better (worse) clinical improvement, higher proportions of hospital discharge or recovery, higher (lower) risk of composite outcomes, compared to the control group

Supplementary Table 5. Subgroup analysis on the outcomes

	Before weighting		After weighting		
	Metformin	Control	Metformin vs Control		
	% (N)	% (N)	OR†	95% CI	P-value
**Clinical improvement on WHO clinical progression scale by ≥ 1 score**					
Overall	97.1% (786)	80.4% (428)	2.74	(1.31, 5.71)	0.009
Age ≤ 65	99.5% (418)	94.5% (183)	4.57	(1.19, 17.51)	0.027
Age > 65	94.3% (368)	69.8% (245)	2.75	(1.24, 6.11)	0.015
Male	96.9% (423)	77.2% (237)	4.55	(1.43, 14.51)	0.013
Female	97.2% (363)	84.3% (191)	1.26	(0.55, 2.89)	0.579
Without invasive mechanical ventilation or ECMO	97.1% (780)	80.0% (419)	2.67	(1.28, 5.55)	0.010
With invasive mechanical ventilation or ECMO	100.0% (6)	100.0% (9)	NA	NA	NA
Without ICU admission	98.1% (682)	86.3% (278)	2.19	(0.79, 6.04)	0.125
With ICU admission	90.4% (104)	69.3% (150)	5.12	(2.73, 9.60)	<0.001
Without concomitant use of insulin	99.7% (369)	91.4% (151)	5.14	(0.98, 26.92)	0.052
With concomitant use of insulin	94.7% (417)	74.4% (277)	2.50	(1.12, 5.60)	0.027
Without concomitant use of SU	98.1% (413)	80.6% (371)	4.33	(2.27, 8.28)	<0.001
With concomitant use of SU	96.0% (373)	78.9% (57)	1.94	(0.56, 6.66)	0.279
Without concomitant use of remdesivir	98.6% (579)	81.1% (307)	4.78	(1.33, 17.09)	0.018
With concomitant use of remdesivir	92.8% (207)	78.5% (121)	1.14	(0.55, 2.38)	0.717
Without concomitant use of interferon-β-1b	98.6% (283)	88.5% (122)	1.50	(0.37, 6.17)	0.561
With concomitant use of interferon-β-1b	96.2% (503)	77.1% (306)	3.21	(1.36, 7.56)	0.010
Without concomitant use of dexamethasone	99.8% (445)	93.1% (173)	6.58	(1.16, 37.46)	0.034
With concomitant use of dexamethasone	93.5% (341)	71.8% (255)	3.00	(1.45, 6.23)	0.004
**Hospital discharge (score ≤ 3)**					
Overall	95.7% (786)	76.6% (428)	2.26	(1.24, 4.12)	0.009
Age ≤ 65	98.3% (418)	92.9% (183)	1.82	(0.77, 4.28)	0.170
Age > 65	92.7% (368)	64.5% (245)	2.41	(1.21, 4.82)	0.015
Male	96.0% (423)	72.6% (237)	3.45	(1.41, 8.44)	0.008
Female	95.3% (363)	81.7% (191)	1.14	(0.55, 2.34)	0.722
Without invasive mechanical ventilation or ECMO	95.9% (780)	77.1% (419)	2.29	(1.22, 4.31)	0.012
With invasive mechanical ventilation or ECMO	66.7% (6)	55.6% (9)	5.30	(1.02, 27.56)	0.047
Without ICU admission	97.2% (682)	84.5% (278)	2.05	(0.83, 5.06)	0.116
With ICU admission	85.6% (104)	62.0% (150)	3.22	(1.86, 5.55)	<0.001
Without concomitant use of insulin	98.9% (369)	90.7% (151)	2.84	(0.93, 8.67)	0.067
With concomitant use of insulin	92.8% (417)	69.0% (277)	2.12	(1.09, 4.14)	0.028
Without concomitant use of SU	96.9% (413)	76.5% (371)	3.00	(1.87, 4.83)	<0.001
With concomitant use of SU	94.4% (373)	77.2% (57)	1.70	(0.57, 5.10)	0.330
Without concomitant use of remdesivir	97.6% (579)	79.2% (307)	3.66	(1.32, 10.15)	0.015
With concomitant use of remdesivir	90.3% (207)	70.2% (121)	1.15	(0.63, 2.09)	0.653
Without concomitant use of interferon-β-1b	97.9% (283)	86.9% (122)	1.32	(0.41, 4.22)	0.628
With concomitant use of interferon-β-1b	94.4% (503)	72.5% (306)	2.56	(1.27, 5.17)	0.010
Without concomitant use of dexamethasone	99.1% (445)	93.1% (173)	2.68	(0.91, 7.90)	0.073

With concomitant use of dexamethasone	91.2% (341)	65.5% (255)	2.61	(1.43, 4.76)	0.003
**Recovery (score ≤ 4)**					
Overall	85.6% (139)	60.1% (173)	2.54	(1.34, 4.80)	0.005
Age ≤ 65	94.1% (51)	81.0% (58)	2.56	(0.80, 8.14)	0.112
Age > 65	80.7% (88)	49.6% (115)	3.08	(1.47, 6.43)	0.004
Male	86.4% (88)	55.3% (114)	3.71	(2.07, 6.67)	<0.001
Female	84.3% (51)	69.5% (59)	1.29	(0.41, 4.11)	0.656
Without invasive mechanical ventilation or ECMO	86.5% (133)	60.4% (164)	2.42	(1.24, 4.72)	0.010
With invasive mechanical ventilation or ECMO	66.7% (6)	55.6% (9)	5.30	(1.02, 27.56)	0.047
Without ICU admission	85.7% (35)	47.8% (23)	1.28	(0.30, 5.42)	0.732
With ICU admission	85.6% (104)	62.0% (150)	3.22	(1.86, 5.55)	<0.001
Without concomitant use of insulin	93.8% (16)	55.6% (9)	17.46	(1.77, 172.70)	0.014
With concomitant use of insulin	84.6% (123)	60.4% (164)	2.29	(1.19, 4.38)	0.014
Without concomitant use of SU	86.0% (57)	60.6% (155)	3.16	(1.68, 5.93)	<0.001
With concomitant use of SU	85.4% (82)	55.6% (18)	1.83	(0.59, 5.67)	0.289
Without concomitant use of remdesivir	88.1% (67)	62.1% (95)	5.28	(2.19, 12.72)	<0.001
With concomitant use of remdesivir	83.3% (72)	57.7% (78)	1.47	(0.64, 3.35)	0.354
Without concomitant use of interferon-β-1b	89.7% (29)	50.0% (26)	1.63	(0.33, 8.00)	0.538
With concomitant use of interferon-β-1b	84.5% (110)	61.9% (147)	3.05	(1.78, 5.21)	<0.001
Without concomitant use of dexamethasone	95.7% (23)	68.4% (19)	3.84	(0.54, 27.35)	0.178
With concomitant use of dexamethasone	83.6% (116)	59.1% (154)	2.56	(1.30, 5.05)	0.008
**In-hospital death (score = 10)**					
Overall	3.6% (786)	20.3% (428)	0.41	(0.22, 0.80)	0.010
Age ≤ 65	0.7% (418)	6.0% (183)	0.36	(0.12, 1.10)	0.074
Age > 65	6.8% (368)	31.0% (245)	0.40	(0.19, 0.84)	0.017
Male	3.5% (423)	24.1% (237)	0.25	(0.09, 0.70)	0.010
Female	3.6% (363)	15.7% (191)	0.92	(0.43, 2.00)	0.838
Without invasive mechanical ventilation or ECMO	3.3% (780)	19.8% (419)	0.40	(0.20, 0.81)	0.013
With invasive mechanical ventilation or ECMO	33.3% (6)	44.4% (9)	0.19	(0.04, 0.98)	0.047
Without ICU admission	2.3% (682)	14.7% (278)	0.48	(0.19, 1.24)	0.125
With ICU admission	11.5% (104)	30.7% (150)	0.26	(0.15, 0.47)	<0.001
Without concomitant use of insulin	0.5% (369)	9.3% (151)	0.24	(0.06, 0.97)	0.045
With concomitant use of insulin	6.2% (417)	26.4% (277)	0.45	(0.22, 0.93)	0.033
Without concomitant use of SU	2.7% (413)	20.5% (371)	0.29	(0.17, 0.49)	<0.001
With concomitant use of SU	4.6% (373)	19.3% (57)	0.56	(0.17, 1.83)	0.322
Without concomitant use of remdesivir	1.7% (579)	19.2% (307)	0.22	(0.07, 0.73)	0.016
With concomitant use of remdesivir	8.7% (207)	23.1% (121)	1.04	(0.54, 2.00)	0.899
Without concomitant use of interferon-β-1b	1.8% (283)	10.7% (122)	0.87	(0.26, 2.95)	0.818
With concomitant use of interferon-β-1b	4.6% (503)	24.2% (306)	0.35	(0.16, 0.75)	0.009
Without concomitant use of dexamethasone	0.2% (445)	6.4% (173)	0.16	(0.03, 0.94)	0.042
With concomitant use of dexamethasone	7.9% (341)	29.8% (255)	0.38	(0.20, 0.72)	0.004
**Acidosis**					
Overall	1.1% (786)	0.9% (428)	6.82	(2.56, 18.18)	<0.001

Age ≤ 65	1.2% (418)	0.0% (183)	NA	NA	NA
Age > 65	1.1% (368)	1.6% (245)	6.06	(2.11, 17.40)	<0.001
Male	1.7% (423)	1.3% (237)	2.48	(0.81, 7.57)	0.109
Female	0.6% (363)	0.5% (191)	26.15	(3.94, 173.41)	<0.001
Without invasive mechanical ventilation or ECMO	0.9% (780)	1.0% (419)	2.06	(0.73, 5.86)	0.174
With invasive mechanical ventilation or ECMO	33.3% (6)	0.0% (9)	NA	NA	NA
Without ICU admission	0.0% (682)	0.0% (278)	NA	NA	NA
With ICU admission	8.7% (104)	2.7% (150)	7.43	(2.76, 19.99)	<0.001
Without concomitant use of insulin	0.0% (369)	0.0% (151)	NA	NA	NA
With concomitant use of insulin	2.2% (417)	1.4% (277)	7.74	(2.82, 21.25)	<0.001
Without concomitant use of SU	1.5% (413)	0.8% (371)	8.38	(2.69, 26.08)	<0.001
With concomitant use of SU	0.8% (373)	1.8% (57)	3.16	(0.51, 19.42)	0.214
Without concomitant use of remdesivir	0.9% (579)	1.3% (307)	6.19	(2.25, 17.01)	<0.001
With concomitant use of remdesivir	1.9% (207)	0.0% (121)	NA	NA	NA
Without concomitant use of interferon-β-1b	0.7% (283)	0.8% (122)	0.91	(0.13, 6.19)	0.923
With concomitant use of interferon-β-1b	1.4% (503)	1.0% (306)	10.28	(3.40, 31.10)	<0.001
Without concomitant use of dexamethasone	0.4% (445)	0.6% (173)	1.62	(0.26, 10.02)	0.603
With concomitant use of dexamethasone	2.1% (341)	1.2% (255)	9.16	(2.98, 28.16)	<0.001
**Macrophage activation**					
Overall	30.4% (786)	45.1% (428)	0.74	(0.59, 0.93)	0.009
Age ≤ 65	28.5% (418)	36.6% (183)	0.72	(0.52, 0.98)	0.040
Age > 65	32.6% (368)	51.4% (245)	0.77	(0.56, 1.06)	0.109
Male	38.3% (423)	52.7% (237)	0.79	(0.58, 1.07)	0.129
Female	21.2% (363)	35.6% (191)	0.67	(0.45, 1.00)	0.050
Without invasive mechanical ventilation or ECMO	30.1% (780)	44.4% (419)	0.69	(0.55, 0.87)	0.002
With invasive mechanical ventilation or ECMO	66.7% (6)	77.8% (9)	3.42	(0.43, 27.09)	0.244
Without ICU admission	25.8% (682)	28.1% (278)	0.78	(0.58, 1.05)	0.104
With ICU admission	60.6% (104)	76.7% (150)	0.42	(0.26, 0.68)	<0.001
Without concomitant use of insulin	15.2% (369)	17.2% (151)	0.58	(0.37, 0.90)	0.015
With concomitant use of insulin	43.9% (417)	60.3% (277)	0.90	(0.66, 1.21)	0.471
Without concomitant use of SU	28.6% (413)	45.0% (371)	0.73	(0.55, 0.95)	0.021
With concomitant use of SU	32.4% (373)	45.6% (57)	0.77	(0.47, 1.28)	0.310
Without concomitant use of remdesivir	24.2% (579)	37.5% (307)	0.88	(0.66, 1.16)	0.362
With concomitant use of remdesivir	47.8% (207)	64.5% (121)	0.40	(0.27, 0.59)	<0.001
Without concomitant use of interferon-β-1b	18.7% (283)	26.2% (122)	0.73	(0.44, 1.22)	0.230
With concomitant use of interferon-β-1b	37.0% (503)	52.6% (306)	0.76	(0.57, 1.03)	0.075
Without concomitant use of dexamethasone	12.8% (445)	17.3% (173)	0.39	(0.26, 0.60)	<0.001
With concomitant use of dexamethasone	53.4% (341)	63.9% (255)	0.93	(0.69, 1.25)	0.627
**Haematological dysfunction**					
Overall	27.5% (745)	53.4% (388)	0.77	(0.55, 1.10)	0.142
Age ≤ 65	19.4% (407)	40.8% (169)	0.71	(0.49, 1.03)	0.070
Age > 65	37.3% (338)	63.0% (219)	0.86	(0.50, 1.47)	0.564
Male	34.3% (399)	59.4% (207)	0.79	(0.51, 1.21)	0.266
Female	19.7% (346)	46.4% (181)	0.68	(0.47, 1.00)	0.049

Without invasive mechanical ventilation or ECMO	27.5% (743)	52.4% (380)	0.78	(0.55, 1.11)	0.165
With invasive mechanical ventilation or ECMO	50.0% (2)	100.0% (8)	NA	NA	NA
Without ICU admission	20.2% (660)	32.3% (260)	0.82	(0.54, 1.25)	0.348
With ICU admission	84.7% (85)	96.1% (128)	0.19	(0.07, 0.50)	<0.001
Without concomitant use of insulin	10.2% (361)	14.3% (147)	1.82	(0.99, 3.33)	0.053
With concomitant use of insulin	43.8% (384)	77.2% (241)	0.67	(0.41, 1.08)	0.094
Without concomitant use of SU	24.6% (398)	53.4% (337)	0.69	(0.51, 0.93)	0.016
With concomitant use of SU	30.8% (347)	52.9% (51)	0.94	(0.50, 1.75)	0.828
Without concomitant use of remdesivir	20.6% (559)	44.2% (278)	0.69	(0.44, 1.08)	0.105
With concomitant use of remdesivir	48.4% (186)	76.4% (110)	0.92	(0.60, 1.42)	0.711
Without concomitant use of interferon-β-1b	14.4% (271)	25.0% (116)	1.10	(0.69, 1.77)	0.687
With concomitant use of interferon-β-1b	35.0% (474)	65.4% (272)	0.70	(0.46, 1.06)	0.090
Without concomitant use of dexamethasone	4.1% (438)	12.0% (166)	1.00	(0.56, 1.82)	0.987
With concomitant use of dexamethasone	60.9% (307)	84.2% (222)	0.48	(0.33, 0.70)	<0.001
**Coagulopathy**					
Overall	6.1% (783)	15.8% (425)	0.81	(0.58, 1.14)	0.234
Age ≤ 65	3.4% (417)	10.9% (183)	0.46	(0.25, 0.85)	0.013
Age > 65	9.3% (366)	19.4% (242)	1.03	(0.67, 1.59)	0.878
Male	7.9% (420)	19.5% (236)	0.89	(0.58, 1.34)	0.565
Female	4.1% (363)	11.1% (189)	0.67	(0.37, 1.23)	0.194
Without invasive mechanical ventilation or ECMO	5.9% (777)	15.4% (416)	0.83	(0.58, 1.18)	0.291
With invasive mechanical ventilation or ECMO	33.3% (6)	33.3% (9)	0.17	(0.03, 1.02)	0.052
Without ICU admission	2.9% (682)	4.7% (278)	1.35	(0.71, 2.58)	0.357
With ICU admission	27.7% (101)	36.7% (147)	0.58	(0.37, 0.92)	0.020
Without concomitant use of insulin	1.6% (369)	2.6% (151)	1.57	(0.48, 5.13)	0.457
With concomitant use of insulin	10.1% (414)	23.0% (274)	0.83	(0.58, 1.18)	0.307
Without concomitant use of SU	4.4% (412)	15.8% (368)	0.55	(0.35, 0.87)	0.011
With concomitant use of SU	8.1% (371)	15.8% (57)	1.49	(0.83, 2.66)	0.179
Without concomitant use of remdesivir	4.0% (579)	13.4% (306)	0.79	(0.48, 1.31)	0.358
With concomitant use of remdesivir	12.3% (204)	21.8% (119)	0.77	(0.45, 1.29)	0.317
Without concomitant use of interferon-β-1b	2.8% (283)	12.4% (121)	0.45	(0.20, 0.98)	0.043
With concomitant use of interferon-β-1b	8.0% (500)	17.1% (304)	0.98	(0.67, 1.45)	0.935
Without concomitant use of dexamethasone	2.2% (445)	4.0% (173)	1.50	(0.62, 3.59)	0.367
With concomitant use of dexamethasone	11.2% (338)	23.8% (252)	0.70	(0.48, 1.03)	0.067
**Hepatic inflammation**					
Overall	23.0% (738)	47.3% (383)	0.50	(0.38, 0.65)	<0.001
Age ≤ 65	20.4% (393)	40.5% (168)	0.49	(0.32, 0.75)	0.001
Age > 65	26.1% (345)	52.6% (215)	0.50	(0.30, 0.85)	0.012
Male	25.8% (387)	50.2% (205)	0.45	(0.32, 0.63)	<0.001
Female	19.9% (351)	43.8% (178)	0.56	(0.37, 0.84)	0.006
Without invasive mechanical ventilation or ECMO	23.0% (735)	47.1% (382)	0.52	(0.40, 0.67)	<0.001
With invasive mechanical ventilation or ECMO	33.3% (3)	100.0% (1)	NA	NA	NA
Without ICU admission	16.8% (661)	28.5% (270)	0.49	(0.35, 0.68)	<0.001
With ICU admission	76.6% (77)	92.0% (113)	0.15	(0.07, 0.33)	<0.001

Without concomitant use of insulin	12.2% (360)	12.2% (148)	1.02	(0.52, 1.98)	0.959
With concomitant use of insulin	33.3% (378)	69.4% (235)	0.38	(0.23, 0.61)	<0.001
Without concomitant use of SU	18.5% (395)	48.0% (331)	0.47	(0.35, 0.64)	<0.001
With concomitant use of SU	28.3% (343)	42.3% (52)	0.54	(0.32, 0.91)	0.022
Without concomitant use of remdesivir	18.6% (554)	39.3% (280)	0.48	(0.35, 0.68)	<0.001
With concomitant use of remdesivir	36.4% (184)	68.9% (103)	0.49	(0.31, 0.77)	0.003
Without concomitant use of interferon-β-1b	10.7% (270)	22.4% (116)	0.47	(0.29, 0.76)	0.002
With concomitant use of interferon-β-1b	30.1% (468)	58.1% (267)	0.49	(0.34, 0.70)	<0.001
Without concomitant use of dexamethasone	9.0% (434)	15.4% (169)	0.58	(0.32, 1.04)	0.067
With concomitant use of dexamethasone	43.1% (304)	72.4% (214)	0.31	(0.20, 0.47)	<0.001
**Hyperinflammatory syndrome**					
Overall	48.8% (705)	64.3% (353)	0.71	(0.53, 0.95)	0.021
Age ≤ 65	46.2% (385)	52.9% (157)	0.74	(0.53, 1.03)	0.072
Age > 65	51.9% (320)	73.5% (196)	0.70	(0.41, 1.19)	0.183
Male	56.7% (372)	68.3% (183)	0.63	(0.45, 0.88)	0.008
Female	39.9% (333)	60.0% (170)	0.75	(0.51, 1.10)	0.138
Without invasive mechanical ventilation or ECMO	48.8% (705)	64.2% (352)	0.71	(0.54, 0.95)	0.021
With invasive mechanical ventilation or ECMO	NA(0)	100.0% (1)	NA	NA	NA
Without ICU admission	43.9% (636)	51.0% (255)	0.72	(0.54, 0.96)	0.027
With ICU admission	94.2% (69)	99.0% (98)	0.14	(0.02, 1.04)	0.055
Without concomitant use of insulin	32.9% (350)	31.3% (144)	0.88	(0.60, 1.29)	0.509
With concomitant use of insulin	64.5% (355)	87.1% (209)	0.65	(0.37, 1.15)	0.133
Without concomitant use of SU	46.1% (380)	63.5% (304)	0.84	(0.64, 1.10)	0.202
With concomitant use of SU	52.0% (325)	69.4% (49)	0.54	(0.31, 0.94)	0.030
Without concomitant use of remdesivir	42.7% (534)	54.7% (258)	0.85	(0.62, 1.17)	0.312
With concomitant use of remdesivir	67.8% (171)	90.5% (95)	0.21	(0.11, 0.40)	<0.001
Without concomitant use of interferon-β-1b	36.6% (262)	37.8% (111)	1.32	(0.89, 1.96)	0.169
With concomitant use of interferon-β-1b	56.0% (443)	76.4% (242)	0.49	(0.32, 0.76)	0.002
Without concomitant use of dexamethasone	27.5% (426)	30.2% (162)	0.67	(0.46, 0.96)	0.032
With concomitant use of dexamethasone	81.4% (279)	93.2% (191)	0.45	(0.25, 0.81)	0.008

Note: CI = confidence interval; Ct = cycle threshold; ECMO = Extracorporeal membrane oxygenation; OR = odds ratio; ICU = Intensive Care Unit; IgG = Immunoglobulin G; SU = Sulfonylureas

† OR >1 (or <1) indicates metformin group was associated with better (worse) clinical improvement, higher proportions of hospital discharge or recovery, higher (lower) risk of composite outcomes, compared to the control group
